# Stimuli‐Responsive MXene/PNIPAM Hydrogel WITH High‐Performance and Tunable Electromagnetic Interference Shielding Performance

**DOI:** 10.1002/advs.202505551

**Published:** 2025-05-30

**Authors:** Qian Yan, Zonglin Liu, Jinhua Xiong, Huanxin Lian, He Chen, Teng Fei, Yunxiang Chen, Haowen Zheng, Xu Zhao, Liangliang Xu, Fuhua Xue, Yesheng Zhong, Xiaoliang Ma, Liping Shi, Qingyu Peng, Xiaodong He

**Affiliations:** ^1^ National Key Laboratory of Science and Technology on Advanced Composites in Special Environments Center for Composite Materials and Structures Harbin Institute of Technology Harbin 150080 P. R. China; ^2^ Suzhou Research Institute of HIT Suzhou 215104 China

**Keywords:** electromagnetic interference shielding, MXene‐based hydrogel, smart materials, stimuli response

## Abstract

Smart electromagnetic interference (EMI) shielding materials capable of reversible EMI response hold great promise for application in flexible electromagnetic devices. Here, PPM hydrogels composed of poly (N‐isopropylacrylamide) (PNIPAM) and MXene/PEDOT: PSS hybrid fillers are fabricated via ice‐templated freeze‐in‐situ polymerization. The anisotropic structural design of the hydrogel enhances its mechanical properties, conductivity, and EMI shielding properties in a specific direction. Furthermore, it can rapidly adjust its internal water content under thermal and light stimulation. The EMI shielding performance of PPM hydrogels can transition from an EMI shielding status (EMI shielding on, >50 dB) to an EM‐wave‐transparent status (EMI shielding off, ≈2 dB) in the X‐band with the dynamic change in water content. Notably, the EMI SE value of PPM hydrogel in the X‐band can be quantitatively adjusted from ≈59.3 to 15.5 dB, demonstrating excellent repeatability and adjustability. A real‐time electrical switching capability in the broad GHz frequency range (8.2–40 GHz) is achieved through dual stimuli‐responsive (temperature‐responsive and light‐responsive) modulation. This study presents a new model for dynamically switchable EMI shielding and reveals the great application potential of smart on/off switchable hydrogel‐based EMI shielding materials as next‐generation multifunctional electronics.

## Graphical abstract

The anisotropic structural design of the hydrogel enhances its mechanical properties, conductivity, and EMI shielding properties in a specific direction. Furthermore, it can quickly adjust its internal water content under thermal and light stimulation. The EMI shielding performance of PPM hydrogels can shift from EMI shielding status to EM‐wave‐transparent status in the X‐band with the dynamic change in water content.

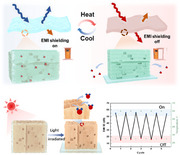

## 1 Introduction

With the advent of the 5G era and the rapid development of the internet, electromagnetic (EM) waves, which play a critical role in high‐density communication environments, have become a double‐edged sword. While electromagnetic waves provide significant benefits to daily life, they also inevitably contribute to electromagnetic pollution and the risk of information leakage, and excessive electromagnetic interference (EMI) or radiation can harm human health^[^ ^1^, ^2^, ^3^ ^]^ and disrupt the normal operation of electronic equipment.^[^ ^4^, ^5^, ^6^ ^]^ Therefore, while fully utilizing the convenience brought by EM waves, it is imperative to achieve comprehensive and intelligent management of the spatial and temporal distribution of EMI fields in the new generation of intelligent EMI shielding materials. Compared with traditional EMI shielding materials with fixed shielding performance,^[^ ^7^, ^8^ ^]^ the new generation of intelligent EMI shielding materials can dynamically regulate their EMI shielding effectiveness (SE) and EMI status by sensing specific application requirements and environmental variations (pressure, pH value, and temperature, etc.),^[^ ^9^, ^10^, ^11^, ^12^ ^]^ which will lead to the widespread application of intelligent EMI shielding materials in complex and high‐precision EMI environments and future smart living.

The deformable conductive network within materials is one of the means to achieve adjustable EMI shielding performance.^[^ ^12^, ^13^, ^14^ ^]^ Existing smart EMI shielding materials adjust the EMI shielding performance by changing the structure of the aerogel conductive network. However, a single method of layer spacing control has a limited tunable range. Compared to aerogels with porous structures, porous hydrogels filled with water molecules with high dielectric loss can simultaneously modulate the structure and component (water,^[^ ^15^ ^]^ ions,^[^ ^16^ ^]^ etc.), thereby enabling a broader tunable range of EMI shielding parameters.

In addition, the stimulating methods are also an important property of smart EMI shielding materials. Compared to smart EMI shielding materials whose electromagnetic parameter variations are triggered under contact stimuli (Strain‐responsive EMI shielding materials,^[^ ^14^, ^17^ ^]^ Chemical reagent‐responsive EMI shielding materials,^[^ ^16^, ^18^ ^]^ etc.), thermosensitive hydrogels can achieve controllable variations in EMI shielding parameters under non‐contact stimuli by incorporating functional materials ^[^ ^19^ ^]^ (photothermal conversion materials such as MXene,^[^ ^20^, ^21^, ^22^, ^23^, ^24^ ^]^ graphene oxide,^[^ ^25^ ^]^ etc.).^[^ ^19^ ^]^ Therefore, applying functional thermosensitive hydrogels in the field of EMI shielding is a crucial innovative direction to address the demands of modern complex EMI environments and multifunctional flexible devices, and is an inevitable trend in the future development of flexible intelligent EMI shielding materials capable of dynamic regulation.^[^ ^26^, ^27^, ^28^ ^]^

Herein, a simple and scalable ice‐templated freezing method was used to prepare a conductive hydrogel with controllable pore morphology (PPM hydrogel). Poly (N‐isopropylacrylamide) (PNIPAM) was used as a flexible thermosensitive substrate, and MXene/PEDOT: PSS ink was used as a conductive filler, mechanical reinforcement, and photothermal agent to form a MXene‐based smart EMI shielding hydrogel via in situ polymerization. The prepared composite hydrogel has a highly ordered and controllable micron‐scale pore morphology inside. The aligned porous structure of the hydrogel not only enhances its mechanical properties but also provides a water transmission channel for the rapid deformation of the hydrogel. Furthermore, the multiple reflections of the incident EM waves are increased by regulating the pores inside the MXene‐based thermosensitive hydrogel, thereby enhancing the EMI shielding performance. Thermosensitive hydrogels exhibit unique thermal responsiveness near the lower critical solution temperature (LCST), resulting in reversible states of water release (T > LCST) and water absorption (T < LCST) under temperature stimulation. By compounding MXene with excellent photothermal conversion efficiency with PNIPAM hydrogel, deformation actuation of the composite hydrogel can be achieved under multiple stimuli such as temperature and light, which enables remote, non‐contact control such as light.

Under the synergistic effect of MXene/PEDOT: PSS, PNIPAM, water, and oriented porous structure, the PPM hydrogel exhibits excellent EMI SE exceeding 50 dB in a broadband frequency range of 8.2‐40 GHz. Owing to its remarkable temperature and light responsiveness of PPM hydrogel, it exhibits excellent reversible and tunable performance in the field of intelligent EMI shielding. With the dynamic change in water content, PPM hydrogels can transition from an EMI shielding status (EMI shielding on, >50 dB) to an EM‐wave‐transparent status (EMI shielding off, ≈2 dB) in X‐band. Upon the temperature rises or light is illuminated, the EMI SE value of PPM hydrogel in the X‐band can be quantitatively adjusted from ≈59.3 to ≈15.5 dB, demonstrating repeatability and adjustability. In addition, the EMI SE value of the hydrogel in the wide GHz frequency range (8.2–40 GHz) can be regulated by controlling the conditions of the external environment. This study has broadened the application of flexible, intelligent, tunable EMI shielding materials in the next generation of multifunctional electronic products and holds significant promise for future technologies.

## 2 Results and Discussion

**Figure 1:**
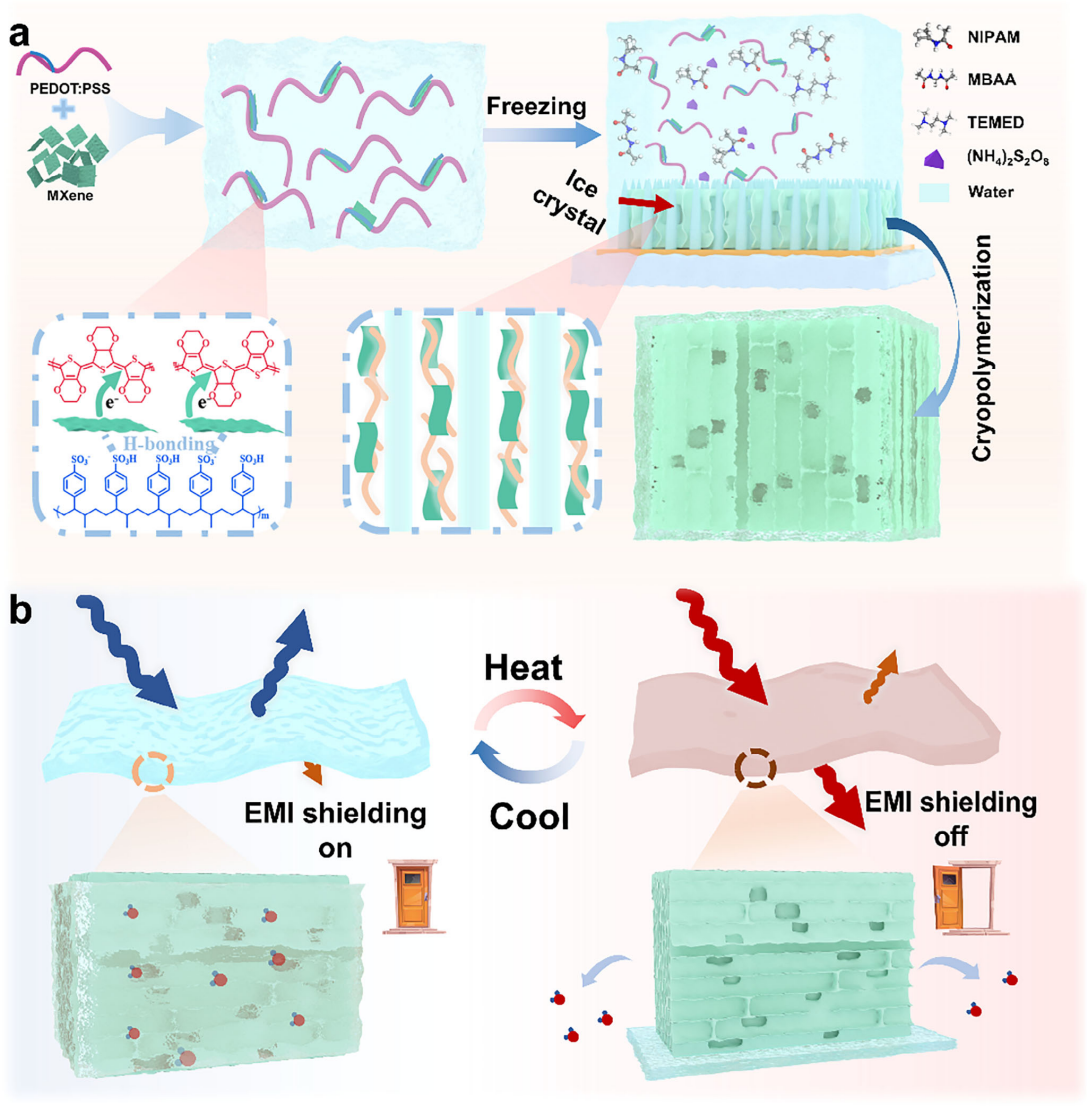
Schematic illustration of stimuli‐responsive hydrogel. a) The schematic illustration of the hydrogel synthesis process. b) Schematic representation of PPM hydrogel with reversible characteristics, illustrating the transition between the EMI shielding and EM‐wave‐transparent states upon heating and cooling.

Herein, we present photothermally modulated EMI shielding hydrogels fabricated via ice‐templated freeze‐casting (**Figure** **1**a). Reversible modulation of the EMI shielding capability is achieved through the synergistic effect of photothermal‐responsive conductive MXene/PEDOT: PSS and thermoresponsive PNIPAM. With the dynamic changes in water content, PPM hydrogels can transition from an EMI shielding state (EMI shielding on, >50 dB) to EM‐wave‐transparent status (EMI shielding off, ≈2 dB) in X‐band (Figure 1b).

### 2.1 Fabrication and Characterization of PPM Hydrogels

The MXene nanosheets and PEDOT: PSS are assembled via electrostatic interaction (Figure S1, Supporting Information; Figure 1a). A monolayer MXene nanosheet, ≈1.8 nm thick with an average lateral size ranging from 1.2 to 2 µm, was synthesized using the MILD method (Figure S2, Supporting Information). The dispersion is mixed with an aqueous dispersion of ink, and the PEDOT molecules with a positive charge interact electrostatically with the negatively charged surface of MXene, thereby preventing the agglomeration of MXene nanosheets. Subsequently, the mixture is then combined with hydrogel monomers for freeze‐oriented in situ copolymerization, forming a cross‐linked hydrogel network. The PPM hydrogel was frozen to produce an internally ordered structure. Ultimately, as the ice crystals melted, an anisotropic hydrogel with an ordered orientation filled with water was obtained. The existence of directional pores inside the hydrogel can accelerate the escape of water, which is primarily due to the capillary effects generated by the ice crystal extrusion constraint inside the polymer and the polymer aggregation caused by the directional open pore structure inside the hydrogel. More importantly, the capillary action facilitates the rapid extrusion and absorption of water molecules, thereby enhancing the deswelling/swelling rate of the anisotropic hydrogel.

**Figure 2:**
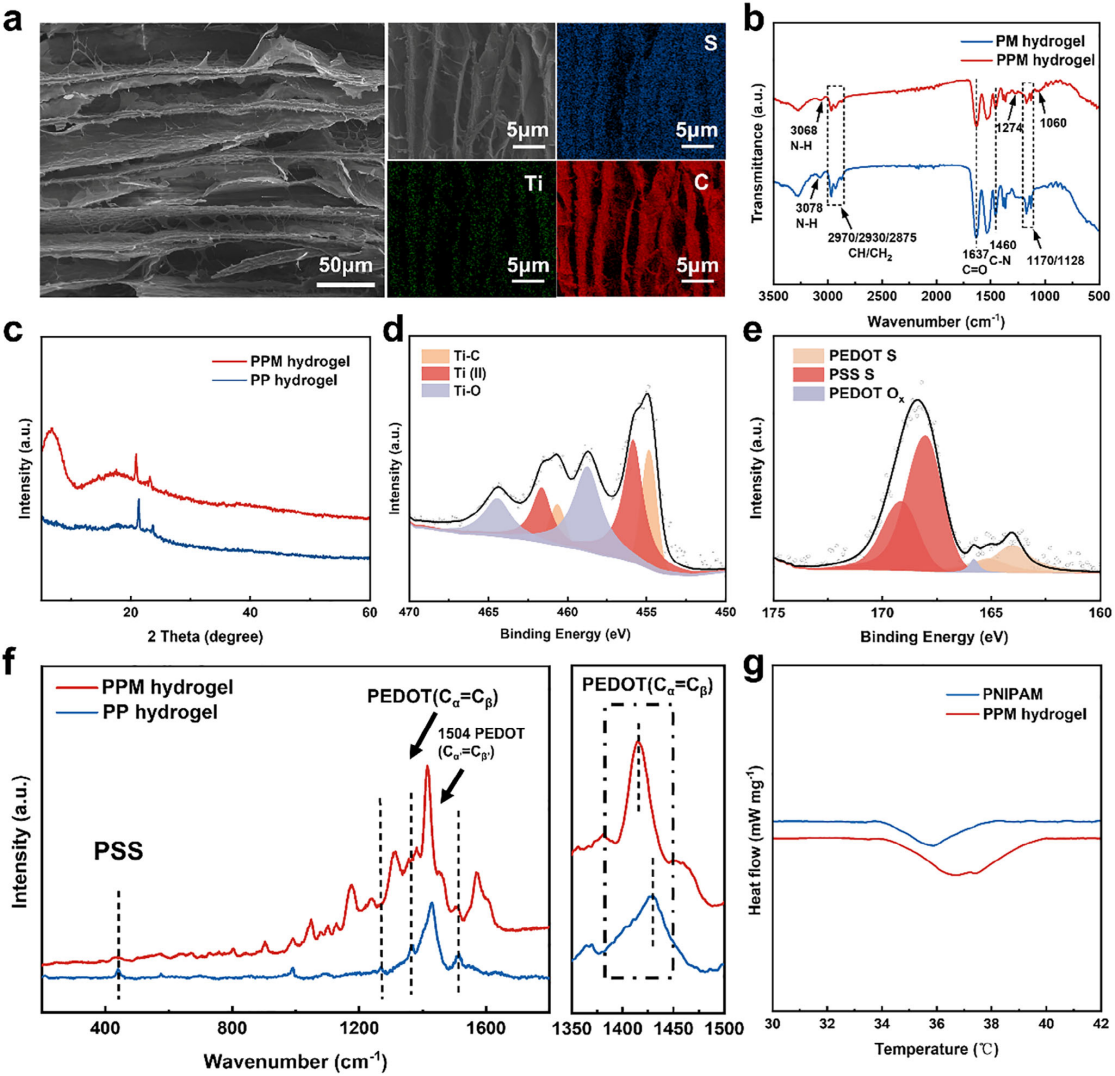
Structural properties and characterization of stimuli‐responsive hydrogel. a) SEM image of PPM hydrogel, accompanied by its corresponding EDS elemental mapping for C, S, and Ti. b) FTIR spectra of the PM and PPM hydrogel; c) The XRD of PP and PPM hydrogel; d) Ti 2p spectra of PPM hydrogel; e) S 2p spectra of PPM hydrogel; f) Raman spectra of PPM hydrogel in comparison with that of PP hydrogel. g) The DSC curves of the pure PNIPAM and PPM hydrogel.

The ordered micron‐scale pores and the distribution of Ti and S elements within the PPM hydrogel, aligned with the direction of ice crystal growth, are clearly observed (**Figure** **2**a). The Fourier transform infrared (FTIR) spectra of PPM hydrogel and MXene/PNIPAM hydrogel (PM hydrogel) are shown in Figure 2b,^[^ ^29^ ^]^ where the characteristic absorption peaks of the CH_2_/CH, C═O, and C─N of PNIPAM are located at 2970/2930/2875 cm^−1^, 1637 cm^−1^ and 1460 cm^−1^, respectively.^[^ ^30^ ^]^ The peak located at 3078 cm^−1^ of PM hydrogel represents the N–H stretching vibration, while this vibration was found to shift to 3068 cm^−1^ in the spectrum of PPM hydrogel, which indicates the existence of hydrogen bonds between PSS and the PNIPAM chains and MXene in the hydrogel system, respectively. Furthermore, it can be observed that peaks corresponding to the ethylenedioxy group appear at ≈1060 and 1270 cm^−1^. Specifically, the characteristic peak stretching vibrations of benzenesulfonate at 1170 and 1128 cm^−1^ indicated the presence of PSS.^[^ ^31^ ^]^ Compared with the PEDOT: PSS/PNIPAM hydrogel (PP hydrogel), the PPM hydrogel with the addition of MXene (Figure S3, Supporting Information) exhibits a (002) diffraction peak. In particular, the (002) diffraction peak is further reduced to 6.71° (Figure 2c), which may be due to the expansion of the interlayer space after the combination of Ti_3_C_2_T_x_ and polymer. Characteristic signals of Ti─C bonds and Ti─O bonds are observed in both Ti_3_C_2_T_x_ and PPM hydrogel (Figure 2d; Figure S4, Supporting Information). In particular, the presence of PEDOT: PSS and PNIPAM chains resulted in a significant enhancement of the Ti‐O peak in the Ti 2p spectrum of the PPM hydrogel (Figure 2d). Besides, the S 2p_3/2_ and S 2p_1/2_ states of the PEDOT chain can be discovered in the PPM hydrogel (Figure 2e).^[^ ^32^ ^]^ Due to the presence of PEDOT: PSS, the Raman spectrum of the PP hydrogel shows that the peak corresponding to the C_α_ = C_β_ symmetric stretching vibration of the five‐membered thiophene ring on PEDOT is located at 1427 cm^−1^, the characteristic band corresponding to the ethylene oxide ring of PEDOT is located at ≈990 cm^−1^, and the characteristic band corresponding to PSS is located at ≈438 cm^−1^ (Figure 2f). It is worth noting that the peak at 1427 cm^−1^ in the PPM hydrogel is red‐shifted by ≈9.83 cm^−1^ due to the transformation of the PEDOT chains from benzoid to quinoid structure caused by strong π‐stacking interaction between Ti_3_C_2_T_x_ and PEDOT (Figure 1a; Figure S5, Supporting Information).^[^ ^33^, ^34^ ^]^

### 2.2 Photothermal‐Responsive Modulated EMI Shielding Performance of PPM Hydrogels

**Figure 3:**
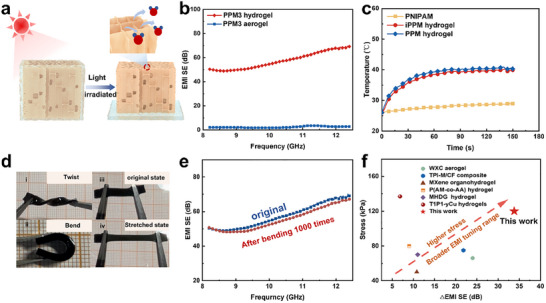
Photothermal‐responsive and mechanical properties of hydrogels. a) Schematic diagram of the PPM hydrogel deswelling process under light exposure; b) X‐band EMI SE of the PPM3 hydrogel and PPM3 aerogel. c) Temperature variation of the iPPM, PPM, and PNIPAM hydrogels under light irradiation at a power density of 0.5 W cm^−2^. d) The photographs of the ultra‐flexible MXene‐based hydrogels bending, twisting, and stretching, e) X‐band EMI SE of PPM3 hydrogels hydrogel before and after 1000 times bending treatment. f) Comparison of the mechanical and tunable EMI shielding range of PPM hydrogel relative to other smart EMI shielding materials.

The PPM hydrogels are composed of a flexible conductive network and water and exhibit varying EMI shielding performance depending on water content. The initial PPM hydrogel in a saturated state shows excellent EMI shielding properties (59.3 dB) (**Figure** **3**b). The thickness of the network shrinks with the escape of water (Figure 3a; Movie S1, Supporting Information), which is beneficial for improving the EMI SE. However, the significant loss of water, which acts as the main EMI loss medium, has masked the gain from the reduction in thickness. Therefore, the PPM aerogel (fully dehydrated state) exhibits an EM‐wave transparency (2.3 dB) (Figure 3b).

The incorporation of MXene, which possesses high photothermal conversion efficiency, into the PPM hydrogel (Figure 3c; Figure S6, Supporting Information) enables phase transitions under continuous light irradiation, facilitating reversible changes in EMI shielding status. The average light absorption of iPPM hydrogel and PPM hydrogel with the incorporation of MXene exceeds 90% (Figure S6a, Supporting Information). Additionally, the temperature variations throughout the irradiation process were recorded by infrared thermal imaging (Figure S6b, Supporting Information). The results show that the temperature rise rate of the composite hydrogel after the introduction of MXene significantly increases. The equilibrium temperature of the PPM hydrogel (Figure S8b, Supporting Information) increased with the increase in light density. Notably, the hydrogel exhibits a heating rate of 1.03 °C s^−1^ at a light power density of 1.7 W cm^−2^, demonstrating the remarkable photothermal conversion capability of PPM hydrogel. In addition, the EMI shielding status of the hydrogel can recover immediately with the interruption of light by swelling (Figure S7, Supporting Information). Therefore, the reversible swelling and deswelling of the PPM hydrogel enables it to achieve a photothermal‐responsive EMI shielding status switch.

The response rate of the electrical switch can be modulated by the intensity of the external stimulus (Figure 3a), the water‐locking ability of hydrogels, and the transmission speed of water, which can be adjusted by the light intensity (Figure S8a,b, Supporting Information), the hydrogels’ critical volume phase transition temperature value (Figure 2g),^[^ ^35^, ^36^ ^]^ and pore structure of the hydrogels (Figure S8c,d, Supporting Information), respectively. Light and temperature are the primary factors regulating the pore structure of temperature‐sensitive hydrogels. Temperature variations can cause the hydrogel to transition from a hydrophilic, expanded state to a hydrophobic, contracted state, thereby regulating the pore structure. More importantly, the deswelling rate of the hydrogel under different light power densities reflects the escape rate of water molecules. The weight of the anisotropic PPM hydrogel deswells quickly to 45% within 4 s (Figure S8c and Movie S2, Supporting Information), and its deswelling rate is substantially higher than isotropic hydrogel (Figure S8d, Supporting Information). Therefore, this further proves that the orderly‐oriented pores facilitate the faster escape of water molecules within the hydrogel. The directional open pore structure inside the PPM hydrogel enables it to provide directional transport channels for water molecules under continuous light irradiation. The reason why the existence of directional pores can accelerate the escape of water is mainly related to the polymer aggregation effect caused by the capillary effect generated by the ice crystal extrusion constraint inside the polymer and the directional open pore structure inside the hydrogel. More importantly, capillary action induces rapid absorption and desorption of water molecules in anisotropic hydrogels. However, in isotropic hydrogels, thermal stimulation causes the outer layer to collapse first, trapping water in disordered internal pores. This dense collapsed layer restricts water escape, resulting in limited deformation (Figure S9, Supporting Information). It is worth noting that the fast response properties of anisotropic hydrogels under light irradiation make them promising candidates in remotely controlled conductive hydrogel applications.

The response rate of the EMI shielding switch is primarily governed by the escape rate of water molecules inside the hydrogel, while the photothermal response rate of the PPM hydrogel is closely related to the directional pore structure inside the hydrogel (Figure 2a). Moreover, the PPM hydrogel exhibits excellent mechanical flexibility, including high bendability, twistability, foldability, and stretchability (Figure 3d; Figure S10, Supporting Information). The shielding performance remains stable after 1000 bending cycles (Figure 3e), indicating that the composite hydrogel has outstanding EMI shielding stability and reliability. Compared with previously reported smart EMI shielding materials, the PPM hydrogel demonstrates a broad tunable EMI shielding range and superior mechanical properties (Figure 3f).^[^ ^17^, ^37^, ^38^, ^39^, ^40^, ^41^ ^]^

Furthermore, the mechanical properties of isotropic and anisotropic hydrogels were compared. The tensile stress and modulus of the isotropic hydrogel were enhanced with the addition of PEDOT: PSS and MXene (Figure S11a, Supporting Information). Notably, the tensile stress of PPM hydrogel is ≈6 times higher than that of the iPPM hydrogel with the same content of MXene and PEDOT: PSS, and even ≈10 times higher than that of the pure PNIPAM hydrogel, with a similar trend observed for modulus. The anisotropic PPM hydrogel fabricated via freeze‐oriented crosslinking has excellent mechanical strength, indicating that the anisotropy of the internal void morphology of the hydrogel forms an enhanced mechanical network in a specific direction, which can effectively disperse and transmit external forces. Also, it has excellent shape adaptability (Figure S10, Supporting Information). Specifically, the tensile stress of the PPM hydrogel can reach 126.43 kPa in the stretching direction parallel to the oriented pores, and the anisotropy ratio of the tensile mechanical properties is ≈1.41 (Figure S11b, Supporting Information). The highly ordered arrangement of the polymer chains and nanosheets in the direction parallel to the pores reduces local stress concentration, thereby resulting in relatively higher strength.

**Figure 4:**
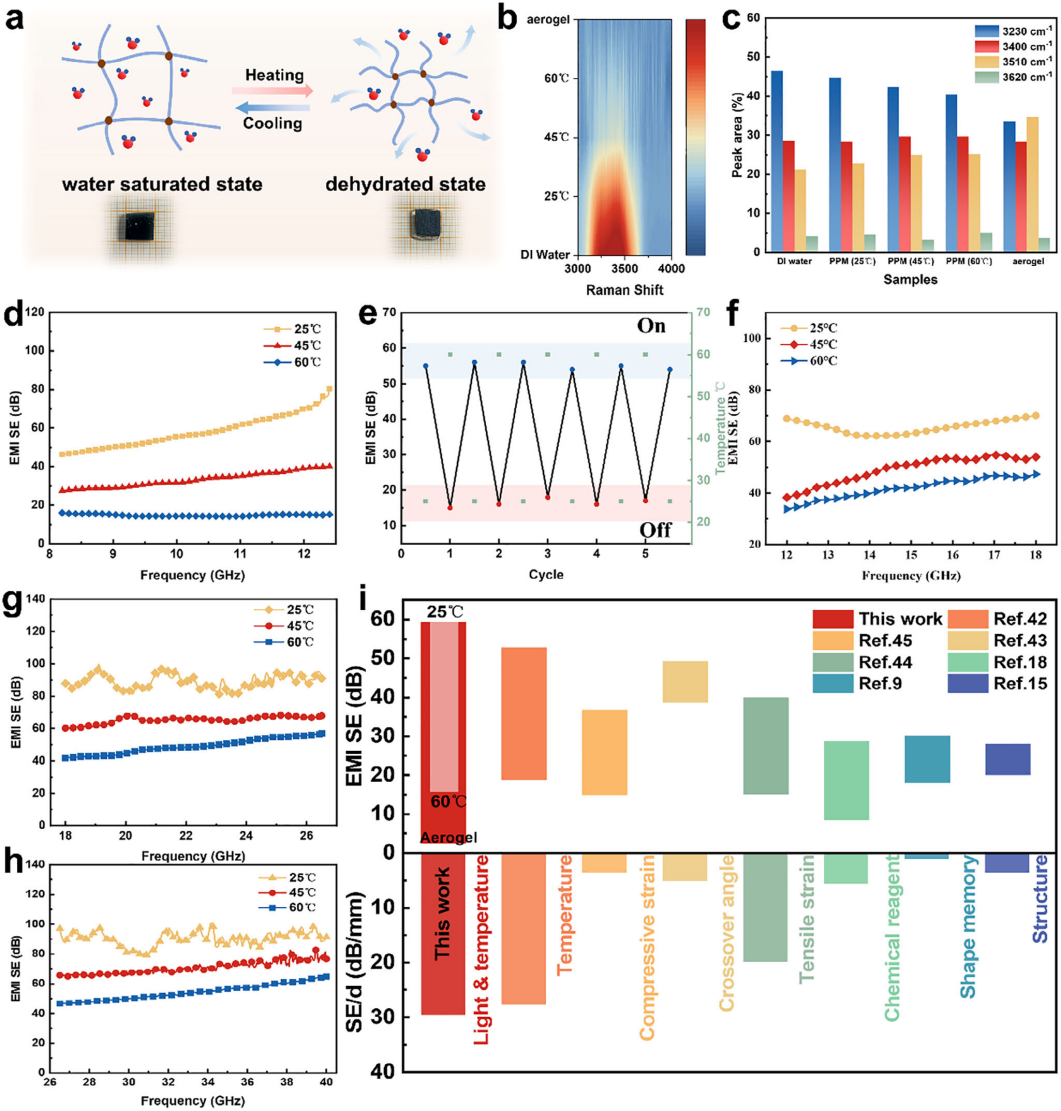
Photothermal‐responsive modulated EMI shielding performance of Hydrogels. a) Schematic diagram and digital photos of hydrogel water loss during the heating process. b) Raman spectrum and c) Peak area of fitting curves of DI water, PPM hydrogel (at 25, 45, and 60 °C), and PPM aerogel. d) The corresponding X band EMI SE curve of PPM3 hydrogel at different temperatures during heating. e) Reversible EMI shielding performance of PPM3 hydrogels with five cycles of heating (60 °C) and cooling (25 °C). f) The corresponding Ku‐band EMI SE curve of PPM3 hydrogel at different temperatures during heating. The corresponding EMI SE curve of PPM hydrogel at different temperatures in g) K‐band (18–26.5 GHz) and h) Ka‐band (26.5–40 GHz) during heating; i) The adjustable shielding effect of existing smart EMI shielding materials under different stimuli.

To track the interactions of the PPM hydrogels with the incident EM waves under different temperatures, a ramp‐up temperature cycle is applied. The phase change behavior of temperature‐sensitive hydrogels causes the mass of water inside to change significantly with temperature (**Figure** **4**a). As the temperature is adjusted from 25 to 60 °C, the PPM hydrogel loses ≈70 wt% of its water (Figure S25a, Supporting Information), while the Raman characteristic peak of water, corresponding to the 2800–3800 cm⁻¹ range, gradually weakens (Figure 4b,c; Figures S12 and S13, Supporting Information). Additionally, the microscopic morphology of the hydrogel at different temperatures is examined. It is observed that, as the temperature increases, the hydrogel shrinks and internally loses water, resulting in a gradual decrease in pore diameter. The hydrogel exhibits a structure of closely packed pores at 60 °C. This occurs because, during the transition to a hydrophobic state, the polymer chains contract, a significant amount of water is expelled, and the pores become smaller or the structure more compact. The results indicate that the hydrogel undergoes a phase transition from a saturated to a dehydrated state as its internal water content changes, which in turn affects its electromagnetic shielding performance. Specifically, the EMI SE of the PPM hydrogel in the X band decreased from 59.3 to 15.5 dB in the X‐band (Figure 4d). When the temperature drops, the hydrogel recovers from the hydrophobic state to the hydrophilic state. During the swelling process, the water content gradually increases, and the EMI SE of the hydrogel returns to its original state (Figure S14, Supporting Information). In particular, the hydrogel exhibits strong temperature dependence and good cycling stability (Figure 4e; Figure S15, Supporting Information), and MXene maintains good stability even after repeated stimulation (Figure S16, Supporting Information). Similarly, PPM hydrogel exhibits a reversible electric switching window of 23.7 dB (65.5 to 41.8 dB, Figure 4f; Figure S17, Supporting Information) in the Ku‐band. In addition, the PPM hydrogel has high electromagnetic shielding performance and excellent stability in the broadband GHz frequency range, which is specifically manifested as 39.81 dB in the K‐band (18–26.5 GHz) (88.89 to 49.08 dB, Figure 4g; Figure S18, Supporting Information) and 32.64 dB in the Ka‐band (26.5–40 GHz) (87.0 to 54.36 dB, Figure 4h; Figure S19, Supporting Information). This shows the universal applicability of smart electromagnetic shielding materials with a tunable range in the broadband GHz frequency range. Compared with other reported smart EMI shielding materials (Figure 4i), the PPM hydrogels offer a rapid electrical switch with a wide adjustable SE range under dual stimulus responses of light irradiation and temperature, which can meet the needs of high‐performance smart EMI shielding that can be remotely controlled.^[^ ^9^, ^15^, ^18^, ^42^, ^43^, ^44^, ^45^ ^]^

### 2.3 Mechanism of the EMI shielding performance of PPM hydrogels

**Figure 5:**
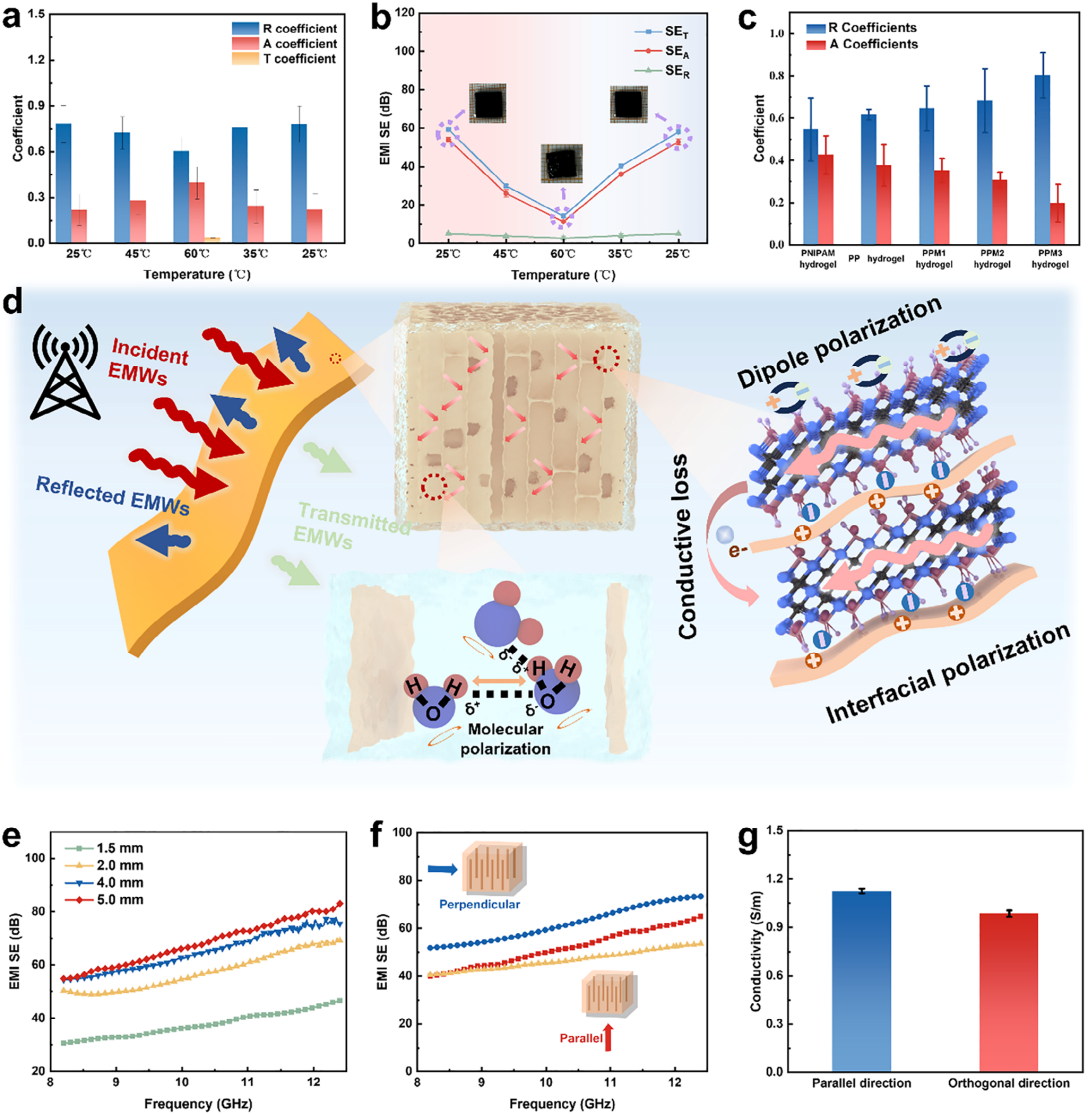
Mechanism and pore‐structure‐domain behavior of the anisotropic hydrogel. The corresponding a) Power coefficients and b) SE_A_, SE_R_, and SE_T_ values in the X‐band of PPM3 hydrogels at different temperatures during heating and cooling. c) Power coefficients of the hydrogels with various MXene contents. d) The mechanism for EMI shielding in hydrogels with aligned pore channels. e) Effect of sample thickness on the EMI SE of PPM3 hydrogels. f) X‐band EMI SE of the PPM3 hydrogels with different pore morphologies. g) The conductivity of PPM3 hydrogel in orthogonal and parallel directions.

To understand the fundamental EM wave responses to the photothermal‐responsive stimulus, the transmission (T), absorption (A), and reflection (R) power coefficients of the PPM hydrogels in the X‐band and Ku‐band during the ramp‐up temperature cycle are calculated (**Figure** **5**a; Figure S20, Supporting Information). During the phase transition of the hydrogel, the compression of the conductive network is beneficial for EMI shielding, while the loss of moisture is harmful. Hence, the EMI shielding performance of PNIPAM hydrogel and PNIPAM aerogel is tested to reveal the main effect on the EMI shielding status of the hydrogels. The PNIPAM aerogel delivers a low R value of 0.01, indicating an EM‐wave transparent performance (Figures S21 and S22, Supporting Information). In contrast, most of the EM waves are reflected first during the propagation process, and then ≈43% of the EM waves penetrate the PNIPAM hydrogel and are absorbed and dissipated by the cell wall and water inside. The strong reflection resulting from the high dielectric constant of water induces an impedance mismatch, which is manifested as R higher than A. Therefore, the high reflection of the incident EM waves of PPM hydrogel is mainly due to the high concentration of water (≈96%) (Figure 5c).

When the temperature exceeds the LCST of the PPM hydrogel, the PPM hydrogel undergoes a phase transition, enhancing the internal hydrophobic effect, which accelerates water loss and leads to a decrease in EMI SE. Simultaneously, the conductivity of the PPM hydrogel also changes accordingly. (Figure S23, Supporting Information). Furthermore, the permittivity of the PPM hydrogel decreases with the escape of water (Figure S24, Supporting Information), and the reduction in the water molecule content inside the PPM hydrogel causes it to exhibit lower tangent loss values (Figure S25b, Supporting Information). Therefore, the R value decreases and the A value increases, indicating more EM waves enter the hydrogel and are dissipated (Figure 5a; Figure S20, Supporting Information).^[^ ^46^ ^]^ However, the hydrogel exhibits a lower SE_T_ value than the initial status due to the decreasing amount of polarization loss caused by water molecules and hydrogen bond networks (Figure 5b; Figure S26, Supporting Information).

The flexible conductive network formed by the copolymerization of MXene, PEDOT: PSS, and PNIPAM plays a crucial role in EMI shielding. The abundant surface functional groups on MXene and the numerous interfaces within the polymer network contribute to significant dipole polarization and interfacial polarization losses. Therefore, the PPM hydrogel possesses a strong EMI shielding capability in its initial saturated state. Furthermore, as the MXene content increases, the EMI shielding performance of the PPM hydrogel improves markedly, enabling tunable EMI shielding performance for practical applications (Figure S27a, Supporting Information). The PPM3 hydrogel exhibited an average EMI shielding effectiveness (SE) of 59.34 dB in the X‐band, effectively blocking over 99.997% of incident EM waves (Figure S27b, Supporting Information). Also, the reflection shielding effectiveness (SE_R_) is primarily influenced by the MXene content (Figure S27c, Supporting Information), which is attributed to the increase in the number of mobile charge carriers in the conductive path, resulting from the higher concentration of MXene nanosheets.^[^ ^47^ ^]^ With increasing MXene content, the absorption shielding effectiveness (SE_A_) of the PPM hydrogel exhibits an upward trend and becomes the dominant contributor to the total shielding effectiveness (SE_T_), consistent with observations in other reported aerogels.^[^ ^48^ ^]^ To reveal the effect of network conductivity on the EMI shielding properties, the amount of MXene is taken as a variable parameter of PPM hydrogels. As the MXene content increases, the conductivity of the network improves correspondingly (Figure S28, Supporting Information). The R value is primarily governed by the impedance difference between two sides of the incident interface, and the shielding layer with higher conductivity will cause an enhancement in the reflection of EM waves.^[^ ^46^ ^]^ Therefore, the R of the PPM hydrogel is increased with the MXene content increases, while the trend of A is opposite (Figure 5c).

According to the above in situ analysis, a schematic of the internal EMI shielding mechanism in PPM hydrogel is shown in Figure 5d. The 3D conductive network filled with abundant water inside the PPM hydrogel (Defined as the water saturated state) imparts excellent EMI shielding properties. The polarization relaxation of water molecules in PPM hydrogel and the changes in the hydrogen bond network under the EMI field lead to the polarization‐induced attenuation of incident waves. In addition, the strong dipolar polarization induced by the interface between the polymer chains and the MXene nanosheets, along with the functional groups therein, contributes to an increase in conductivity loss and polarization loss, thereby enhancing the attenuation and dissipation of EM waves. In addition, the presence of MXene nanosheets improves the mobile carriers and conductivity in the hydrogel, leading to a significant reflection loss of the incident EM waves. Also, the presence of directional channels causes the incident EM waves to be reflected and scattered multiple times, and the propagation path is extended to interact more with the cell wall with high EM wave loss capacity. In particular, with the addition of PEDOT: PSS, the conductivity of the cell wall is further increased, which leads to significantly enhanced reflections and induced EM wave loss due to the biomimetic arrangement of the porous structure of the hydrogel. Based on the synergistic effect of the above conductive consumption, polarization consumption, and multiple reflections, the PPM hydrogel in the saturated state has an excellent anisotropic EMI shielding performance.

Moreover, the combination of MXene, with its high photothermal conversion efficiency, and PNIPAM, with its temperature‐responsive phase transition behavior, enables reversible modulation of water content within the hydrogel under light or thermal stimuli. Additionally, when the PPM hydrogel is in a dehydrated state, the polarization loss associated with internal water molecules is significantly reduced, and the limited water content diminishes absorption capacity, thereby weakening EMI shielding performance. More importantly, the oriented porous structure imparts the hydrogel with a rapid response rate and excellent mechanical strength, making it an intelligent electromagnetic shielding device with mechanical stability, repeatability, and rapid tunability. Therefore, the water content in the hydrogel can be precisely regulated by controlling the light intensity and temperature, thereby enabling non‐contact quantitative control of the EMI shielding capability of the hydrogel.

### 2.4 Thickness‐Dependent Behavior and Pore‐Structure‐Domain Behavior

Given the critical role of water content in hydrogel and its significant influence on EMI SE, the effect of hydrogel thickness on EMI shielding capability is investigated. The EMI SE of PPM hydrogel at the saturated state increased from 37.62 to 68.22 dB with the hydrogel thickness increasing from 1.5 to 5.0 mm (Figure 5e). The increasing absolute amount of water contributes to a broader switching range of PPM hydrogels. This enhancement arises because a thicker hydrogel layer forces the incident electromagnetic waves to traverse more material, resulting in greater attenuation through increased reflection, absorption, and scattering. Consequently, thicker hydrogels provide more transmission pathways, leading to improved overall EMI shielding performance. However, an excessive thickness might reduce the SE divided by the sample thickness (SE/d) value (Figure S29, Supporting Information). Therefore, the 2‐mm‐thick PPM hydrogel, which exhibits the highest SE/d value, is used for EMI shielding tests.

Additionally, the EMI SE of the PPM hydrogel was evaluated for EM waves incident from different directions to assess the influence of the oriented pore structure on shielding performance (Figure 5f). The results indicate that the EMI SE in the perpendicular direction is higher than that in the parallel direction. The enhanced EMI shielding performance in the specific direction of the anisotropic PPM hydrogel can be explained by the highly ordered arrangement of the polymer backbone and nanosheets in the direction parallel to the pores, forming an anisotropic conductive network (Figure 5g). Furthermore, the X‐band EMI SE of hydrogels with anisotropic porous structures is higher than that of isotropic hydrogels, which is consistent with the behavior of aerogels with anisotropic structures.^[^ ^49^ ^]^ This is because the anisotropic alignment of hydrogels' pores and more cell walls and interfaces are used to repeatedly reflect incident EMW, and the propagation path is extended to interact more with the cell walls that have a high EMW loss capacity. The results show that different pore morphologies can also affect the EMI shielding performance of hydrogels. Smart electromagnetic shielding materials generally require both excellent electromagnetic shielding performance and a satisfactory tunable range. In comparison to other reported hydrogel shielding materials (Table S1, Supporting Information), anisotropic PPM hydrogels exhibit superior electromagnetic shielding performance over both carbon‐based hydrogels and isotropic hydrogels. More importantly, unlike other smart electromagnetic shielding materials that rely on a single response mechanism, PPM hydrogels leverage both thermosensitive and photothermal properties to enable a dual‐response mechanism, providing a broad and tunable electromagnetic shielding range. Furthermore, the PPM hydrogel can be remotely controlled via light, a regulatory feature that is rarely found in previous smart shielding materials.

### 2.5 Smart Response Devices Based on PPM Hydrogels

**Figure 6:**
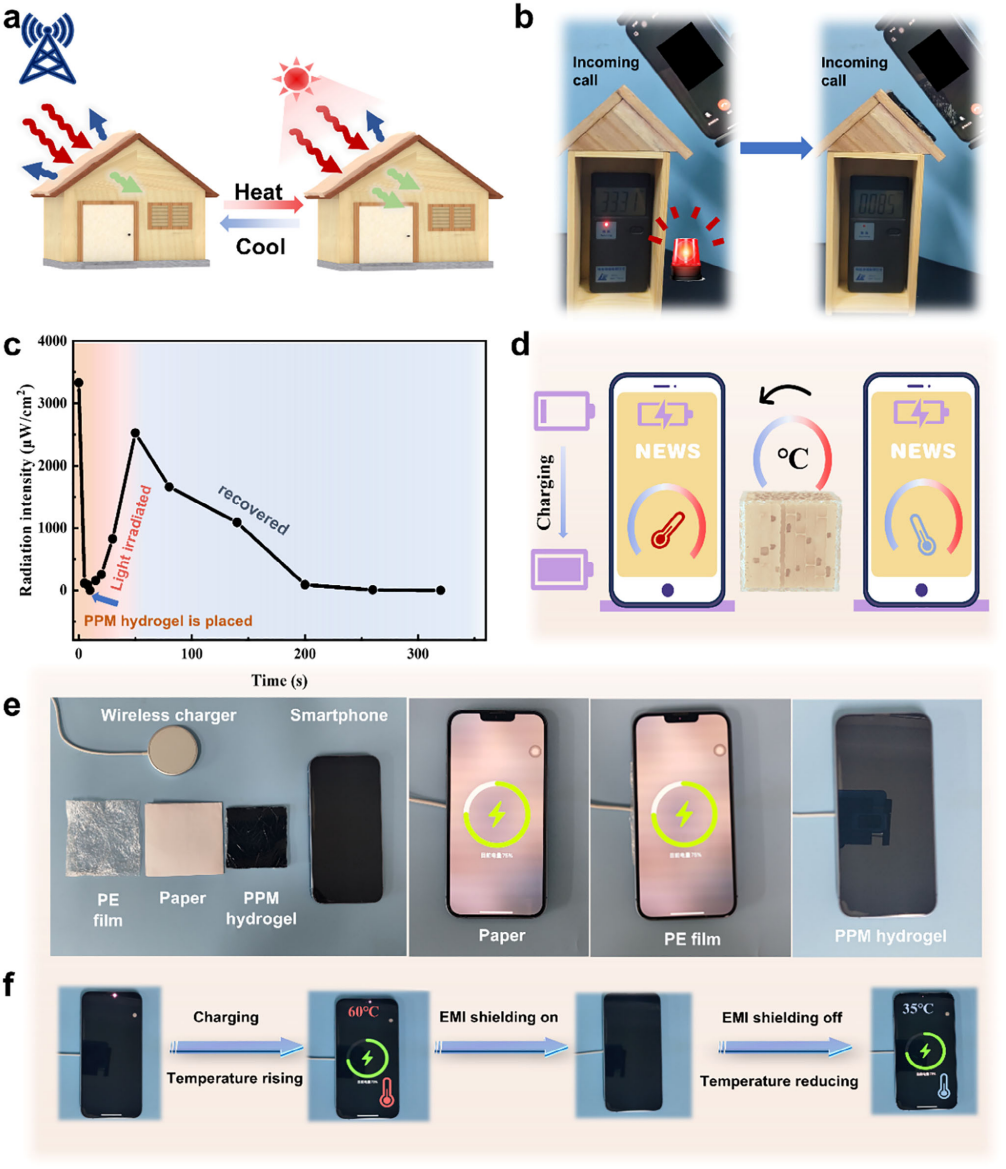
Smart response devices based on PPM hydrogels. a) The schematic of the supposed application of smart hydrogels under light irradiation. b) and c) Reversible electromagnetic shielding effect adjustment of PPM hydrogels on the safe house under light irradiation. d–f) On/off control of the wireless communication by using PPM hydrogels.

Based on the good photothermal‐responsive modulated EMI shielding switch, the PPM hydrogels can be used as intelligent response devices. PPM hydrogels serve as the roof of a room model to assess their light‐responsive electromagnetic (EM) shielding performance (**Figure** **6**a). As shown in Figure 6b,c, the radiation power emitted by a phone call outside is continuously monitored in an unprotected room, with the detected radiation power density measuring 3331 µW cm^−2^, exceeding the radiation limit (200 µW cm^−2^). When the PPM hydrogel was positioned on the roof (EMI shielding on), the radiation power density inside the room dramatically decreased to 85 µW cm^−2^, which is below the radiation limit. Upon exposure to light irradiation, the PPM hydrogel undergoes a phase transition and is in the state of EMI shielding off (Figure 6c; Figure S30, Supporting Information). Consequently, the EM shielding performance weakens gradually, and the radiation power density is increased to 2525 µW cm^−2^. Once the illumination ceased, the radiation power density decreased gradually to 91 µW cm^−2^. This cycle shows that the hydrogel can intelligently regulate the radiation power density inside the room under light response. In future large‐scale applications, the PPM hydrogel will be applied in the form of tiles in house models (Figure S31, Supporting Information), enabling flexibility and scalability in actual application scenarios. This approach provides a novel and effective solution for multi‐functional anti‐radiation intelligent electromagnetic shielding materials in multi‐scene applications. Besides, the EM shielding behavior of PPM hydrogel during wireless charging of smartphones was also studied (Figure 6e; Movie S3, Supporting Information). During the wireless charging process, the mobile phone will inevitably overheat. Once the smartphone overheats, the charging process can be blocked after the PPM hydrogel (EMI shielding on) is placed between the smartphone and the wireless charger (Figure 6d,f). At the same time, the PPM hydrogel undergoes an endothermic phase change behavior under the influence of the overheated smartphone (as a heat source), thereby helping the smartphone to stop charging while also achieving self‐temperature reduction. After the temperature of the smartphone drops, the hydrogel is in an EMI shielding‐off state, allowing the smartphone to continue its charging behavior. The PPM hydrogel offers durability and stability as a smart electromagnetic shielding switch (Figure S32, Supporting Information), providing a novel and effective solution for multifunctional electromagnetic shielding materials across various applications.

## 3 Conclusion

In summary, a thermosensitive PPM hydrogel shielding material is reported in this work, which can reversibly adjust its EMI shielding performance in response to thermal and light stimuli. Anisotropic composite hydrogels are prepared by ice‐templated freezing, and the internally directional aligned open‐pore structure not only makes the hydrogels exhibit excellent mechanical strength and ultraflexibility (bendability, twistability, and even stretchability) but also high conductivity and fast thermal and light response rates. The thin hydrogels show a high EMI SE of ≈59 dB in the X‐band and even have an EMI SE of more than 50 dB in broadband frequency from 8.2 to 40 GHz. As the internal water content dynamically changes, PPM hydrogel can change from a saturated state to an anhydrous state (PPM aerogel), and the EMI value in the X‐band is reduced from >50 dB to ≈2 dB. This corresponds to a transition from the EMI shielding state (EMI shielding on) to the EM wave penetration state (EMI shielding off). Notably, the water content inside the hydrogels can be remotely regulated via multiple external stimuli, and the effect of water on the EMI shielding capability of the hydrogels is quantitatively determined. Therefore, a real‐time electric switch in the wide GHz frequency range (8.2–40 GHz) is achieved through stimuli‐responsive (temperature‐responsive and light‐responsive) modulation. The on/off state of wireless transmission and the radiated power density in a confined space can be controlled by the composite hydrogel through repeated thermal and light stimulation. This smart shielding material with adjustable EMI shielding performance to thermal and light stimulation holds strong potential in smart devices.

## Conflict of Interest

The authors declare no conflict of interest.

## Supporting information



Supporting Information

Supplemental Movie 1

Supplemental Movie 2

Supplemental Movie 3

## Data Availability

Research data are not shared.
