# Supplementary material for: Stimuli‐Responsive MXene/PNIPAM Hydrogel WITH High‐Performance and Tunable Electromagnetic Interference Shielding Performance
Source: Adv Sci (Weinh). 2025 May 30;12(31):e05551. doi: 10.1002/advs.202505551 (PMC12376607; doi:10.1002/advs.202505551)
Supplement: Supplementary file 1 — Supporting Information [file ADVS-12-e05551-s002.docx]

**Supporting Information**

**Stimuli-Responsive MXene/PNIPAM Hydrogel with High-Performance and tunable Electromagnetic Interference Shielding performance**

Qian Yan^1^, Zonglin Liu^1^, Jinhua Xiong^1^, Huanxin Lian^1^, He Chen^1^, Teng Fei^1^, Yunxiang Chen^1^, Haowen Zheng^1^, Xu Zhao^1^, Liangliang Xu^1^, Fuhua Xue^1^, Ye sheng Zhong^1^, Xiaoliang Ma^1^*, Liping Shi^1^*, Qingyu Peng^1,2,^*, Xiaodong He^1^

1. National Key Laboratory of Science and Technology on Advanced Composites in Special Environments, Center for Composite Materials and Structures Harbin Institute of Technology Harbin 150080, P. R. China

2. Suzhou Research Institute of HIT, Suzhou 215104, China

Corresponding author:

Qingyu Peng^1,2^ (email: [pengqingyu@hit.edu.cn](mailto:pengqingyu@hit.edu.cn))

Liping Shi^1^ (email: shiliping@hit.edu.cn)

Xiaoliang Ma^1^ (email: mxlprc@163.com)

**Experimental Section Supplement**

**Materials**

N-Isopropylacrylamide (NIPAM, 98%), N, N, N’, N’-tetramethylethylenediamine (TEMED, 99%), ammonium persulphate (APS, 98%), hexane (97%), Lithium fluoride (LiF, 99%), N, N’-methylenebis (acrylamide) (BIS, 99%) were purchased from Aladdin Shanghai Reagent Co. Ltd. Poly(3,4-ethylenedioxythiophene)/poly(styrenesulfonate) (PEDOT: PSS) were obtained from Heraeus. Ti_3_AlC_2_ MAX powder (400 mesh) and Hydrochloric acid (HCl, 12M) were obtained from Jilin 11 Technology. Co and Ltd and Tianjin Kemiou Chemical Reagent Co., Ltd, respectively.

**Preparation of Ti_3_C_2_T_x_ MXene:**

MXene nanosheets were prepared by the MILD method. Specifically, LiF was added to 40 ml (9 M) hydrochloric acid, and then Ti_3_AlC_2_ powder was slowly added, and the mixture was stirred at 40 °C for 24 hours. It was then rinsed with deionized water 6-9 times to obtain a multilayer MXene precipitate, and the pH value was finally adjusted to about 6. The mixture was placed in a centrifugal container, vortexed for 30 minutes and ultrasonically vibrated for 1 hour, and centrifuged at 1500 rpm for 30 minutes to obtain a supernatant MXene dispersion.

**Preparation of composite hydrogels:**

MXene with different contents was dispersed by ultrasonic stirring in an ice bath for 20 minutes, and then PEDOT: PSS was dispersed in the above solution. Under nitrogen protection, the mixed MXene/PEDOT: PSS mixed solution A was obtained by continuous and vigorous stirring in an ice water bath for 2 hours. N, N'-methylenebis (acrylamide) (BIS) (1 mg) and N-isopropylacrylamide (NIPAM) (100 mg) were added to the mixed solution A in sequence. The mixed solution was bubbled under argon atmosphere for at least 15 minutes to eliminate oxygen. Then, ammonium persulfate (APS) solution was added as an initiator and tetraethylenediamine (TEMED) as a promoter in argon atmosphere. The mixture (abbreviated as M) was then placed in a customized glass mold and polymerized at room temperature for 24 hours under vacuum and oxygen-free conditions to obtain an isotropic iPPM hydrogel. Repeat the above steps to obtain mixture M, then pour the average mixture into a mold (PTFE) with a thick metal base at the bottom, then immerse it in liquid nitrogen for directional freezing, then freeze it in a -20°C refrigerator for 12 hours, and then thaw the frozen hydrogel at room temperature. Repeat the above steps to obtain PPM hydrogel with oriented structure.

**Characterization**

The representation of Ti_3_C_2_T_x_ nanosheets were performed by transmission electron microscopy (TEM, JEM-2100F, 200 kV), atomic force microscope (AFM, Dimension Fastscan, Brucker) and Raman spectrometer in Via-Reflex (RENISHAW) with 532 nm excitation source. The morphology characterization of PPM hydrogel was tested by scanning electron microscopy (SEM, Merlin Compact, Zeiss). Under a nitrogen environment, a temperature ramp of 3.0 °C min^-1^ from 15 to 45 °C was used for the differential scanning calorimetry (DSC, TAQ2000). The light transmittance and reflectance were tested by an ultraviolet-visible near-infrared (UV–vis–NIR) spectrophotometer (UV3600, Shimadzu). An infrared thermal imaging device (VARIOCAM HD880 infrared camera (Infratech)) was used to measure temperature and capture IR thermal pictures. The stress-strength experiments were conducted on a tensile machine (5944, SUNS) with a speed of 10 mm/min. X-ray photoelectron spectroscopy (XPS) and X-ray diffraction (XRD) were carried out by ESCALAB 250Xi (ThermoFisher) and X’Pert Pro (PANalytical), respectively. Fourier transform infrared (FTIR) spectra were performed on a Nicolet iS20 spectrometer.

**Electrical Measurement:**

In order to test the conductivity, the hydrogel sample was placed between copper sheets and the conductivity of the hydrogel was tested using an electrochemical workstation. (50 mV, 106 to 10−1 Hz) (CHI760e). The formula is as follows:

$$\begin{aligned} \sigma=\frac{L}{\left( R \times A \right)}\#(S1) \end{aligned}$$

where L denotes the thickness of the hydrogel, R denotes the measured impedance, and A denotes the tested surface area of the hydrogel.

**EMI shielding Measurement:**

The EMI SE values and electromagnetic parameters in the frequency range of 8.2-40 GHz were measured using a vector network analyzer (VNA, Ceyear 3672C) using the waveguide method. The test sample is placed inside the waveguide cavity, and is heated to different temperatures (calibrate the temperature of the sample using a thermocouple), and test its EMI shielding performance.

The S parameters were recorded and then the total electromagnetic interference shielding effectiveness (SE_T_), reflected electromagnetic interference shielding effectiveness (SE_R_), absorbed electromagnetic interference shielding effectiveness (SE_A_) and the power coefficients of A, R and T were calculated according to the following formulas:^[1]^

$$\begin{aligned} R=\left| S_{11} \right|^{2}, T=\left| S_{21} \right|^{2}\#\left( S2 \right) \end{aligned}$$

$$\begin{aligned} R+A+T=1\#\left( S3 \right) \end{aligned}$$

$$\begin{aligned} SE_{R}=-10\log\left( 1-R \right)\#\left( S4 \right) \end{aligned}$$

$$\begin{aligned} SE_{A}=-10\log\left( \frac{T}{1-R} \right)\#\left( S5 \right) \end{aligned}$$

$$\begin{aligned} SE_{T}=SE_{R}+SE_{A}+SE_{M}\#\left( S6 \right) \end{aligned}$$

**Characterization of Composite Hydrogel**

The Ti_3_C_2_T_x_ MXene nanosheets are obtained by etching the Ti_3_AlC_2_ MAX phase with acid and then layering it by vertexing and oscillation. During the etching process, the Al atomic layer is selectively removed, so that the nanosheets are terminated by surface functional groups (-F, -OH and -O), and a uniformly dispersed MXene dispersion is obtained. As shown in **Figure S4,** the peak at 39° represented the crystal face of aluminum of MAX (Ti_3_AlC_2_) disappeared after the selective etching of Al layer, and the (002) elevation downshifted to 6.96° of Ti_3_C_2_T_x_. MXene monolayer nanosheet with a thickness of about 1.8nm and an average lateral size is about 1.2-2μm is obtained by MILD method (**Figure S2**).

**Electromagnetic Shielding Performance of PPM Hydrogels**

In the saturated state hydrogel, the increase in MXene content will increase the conductivity and thus enhance the EMI SE in the X-band. When the MXene content increases from 0 to 5wt% while keeping the PEDOT: PSS content unchanged, the EMI SE of the composite hydrogel increases accordingly (**Figure S27b**). The PPM3 hydrogel (MXene content 5 wt%) obtained an average X-band SE value of 57.34 dB with more than 99.997% of the incident EM waves blocked. It is worth noting that pure PNIPAM hydrogel without conductive materials also has an EMI SE of ∼15.9 dB. In order to explore its shielding principle, the EMI SE of freeze-dried aerogel was tested to be ∼1 dB (**Figure S21**), which shows the role of internal water in improving EMI SE. The total SET is mainly composed of absorption shielding (SE_A_) and reflection shielding (SE_R_) (**Figure S27c**) In addition, the introduction of MXene and the rich interfaces between polymers and the impedance mismatch in the interface region will lead to high interfacial polarization under the incident EM waves electric field, thereby promoting the absorption of EM waves. At the same time, the rich charge carriers and surface functional groups of the MXene layer also help to improve the dipole polarization loss capacity of the hydrogel. ^[2]^

When the temperature exceeds the LCST of the PPM hydrogel, the PPM hydrogel undergoes a phase transition, enhancing the internal hydrophobic effect, which accelerates water loss and leads to a decrease in EMI SE. Simultaneously, conductivity decreases as the temperature rises (**Figure S23**). This occurs because, as the temperature increases, the hydrogel undergoes a phase transition, causing the conductive network to shift from an ordered layered structure to a spherical one. The increased water loss results in higher interface resistance, which in turn decreases carrier mobility. Consequently, both the reflection and attenuation of electromagnetic waves decrease, leading to a reduction in EMI SE. The EMI SE of PPM hydrogel at saturated state increases from 37.62 to 68.22 dB with the hydrogel thickness increasing from 1.5 mm to 5.0 mm (**Figure 5e**). The decrease in SE/d at a thickness of 5mm may be mainly due to the uneven distribution of water in the hydrogel. In addition, changes in the pore structure limit the contribution of additional thickness to the shielding effect, ^[3]^ resulting in SE/d stopping to increase or even decrease, which is consistent with the behavior of other electromagnetic shielding materials. ^[4]^ The intelligent electromagnetic shielding material of hydrogel can be used as an intelligent switch in wireless charging **(Figure S32a**). The switching process was repeated 50 times (each switching process requires 30 minutes). Moreover, it was used as a radiation power regulation switch and the switching process was repeated 20 times (**Figure S32b**), indicating that it has durability and reliability as an intelligent electromagnetic shielding switch.


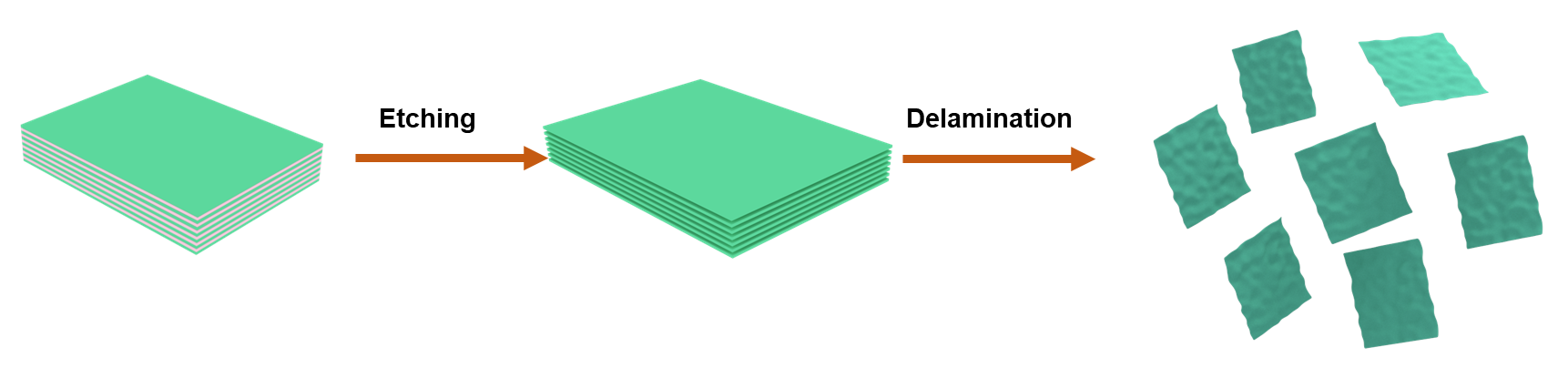


**Figure S1** Schematic illustrating the formation of MXene.


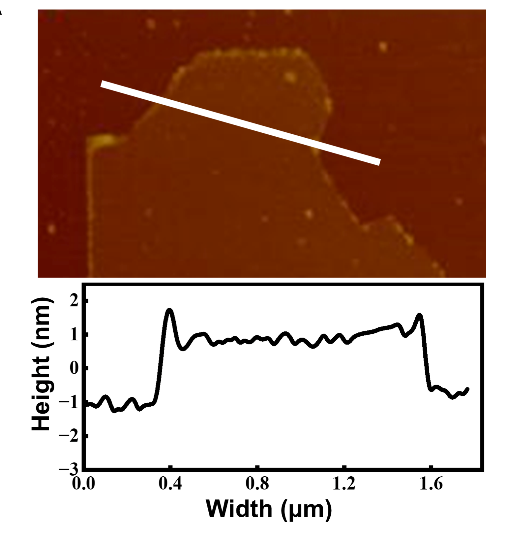


**Figure S2** Atomic force microscope of MXene (Ti_3_C_2_T_x_) nanosheets.


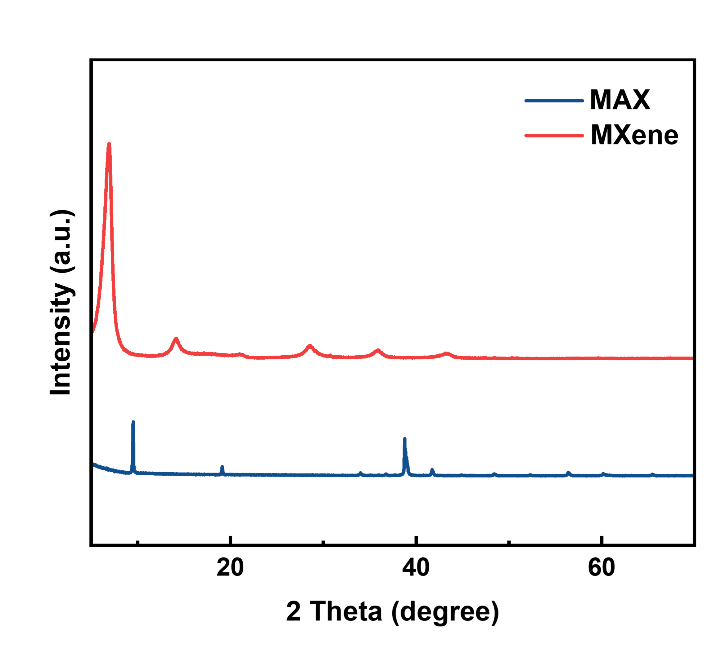


**Figure S3** The XRD of MAX and MXene (Ti_3_C_2_T_x_).


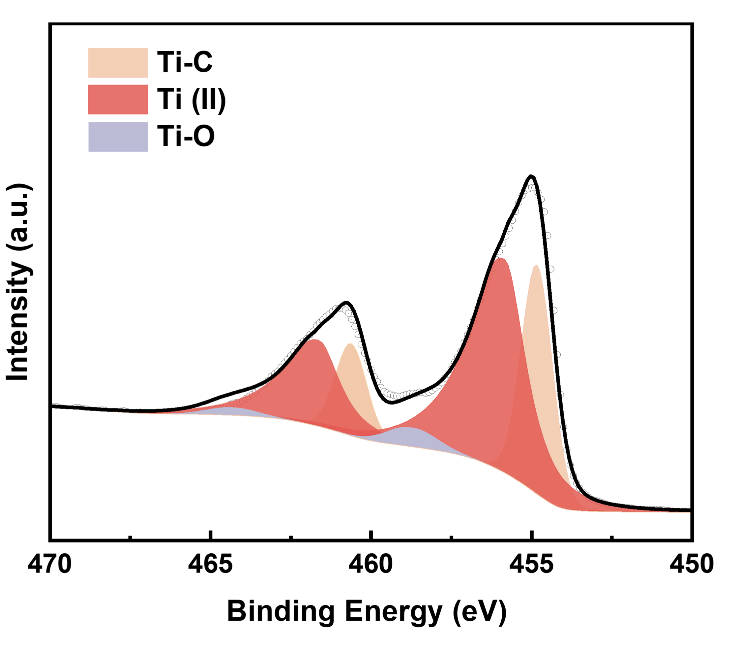


**Figure S4** Ti 2p spectra of Ti_3_C_2_T_x_.


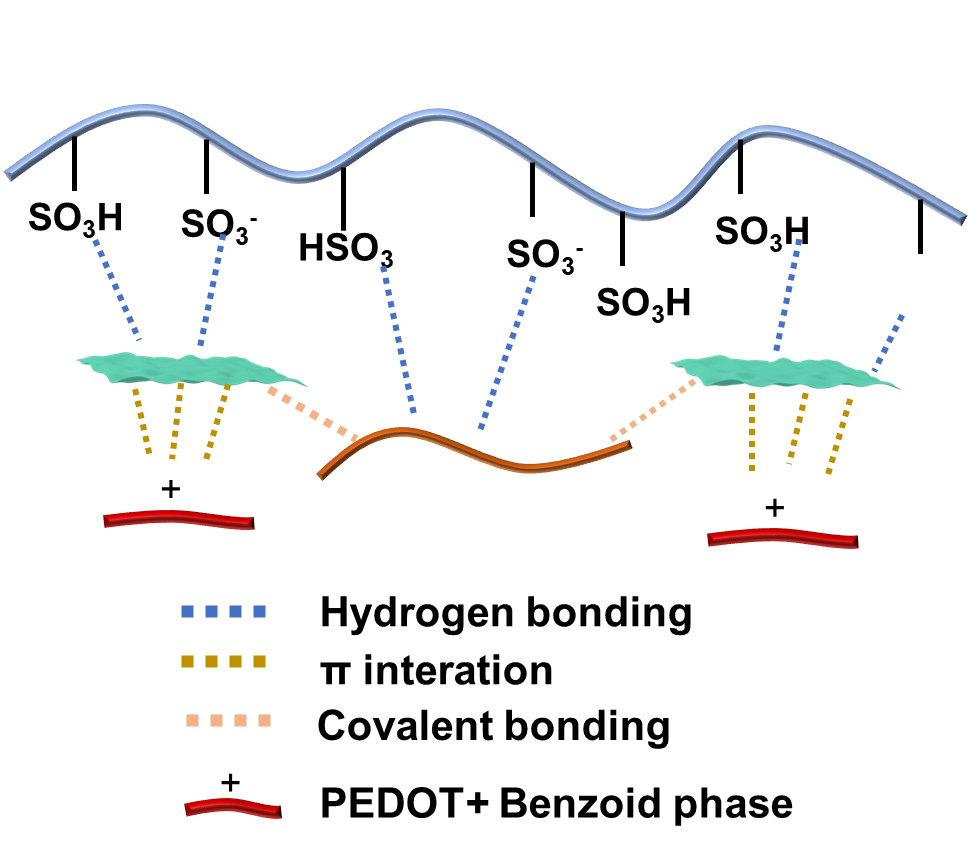


**Figure S5** The molecular-level interaction that occurred between MXene, PEDOT: PSS, and PNIPAM.


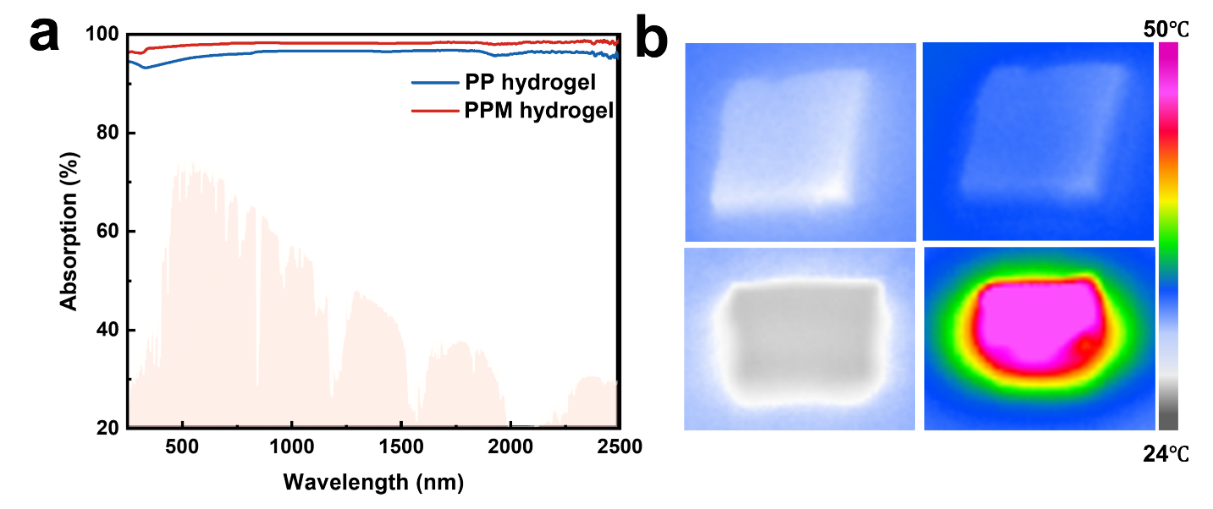


**Figure S6** a) The light absorption of the PP and PPM hydrogels; b) Infrared image of the pure PNIPAM (top side) and PPM hydrogels (bottom side) irradiated light (0.5 W cm^−2^).


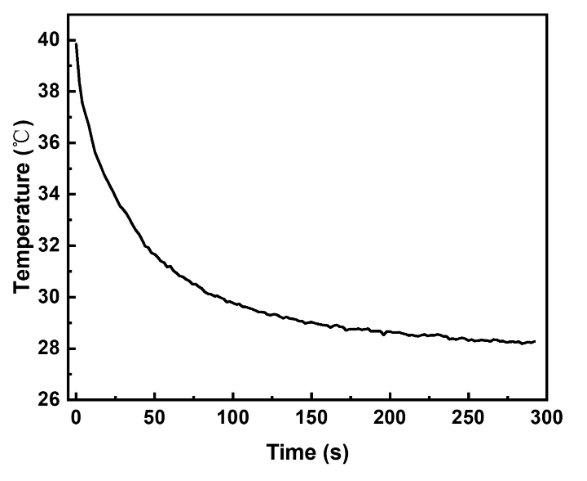


**Figure S7** Temperature evolution in the cooling process of PPM hydrogel.


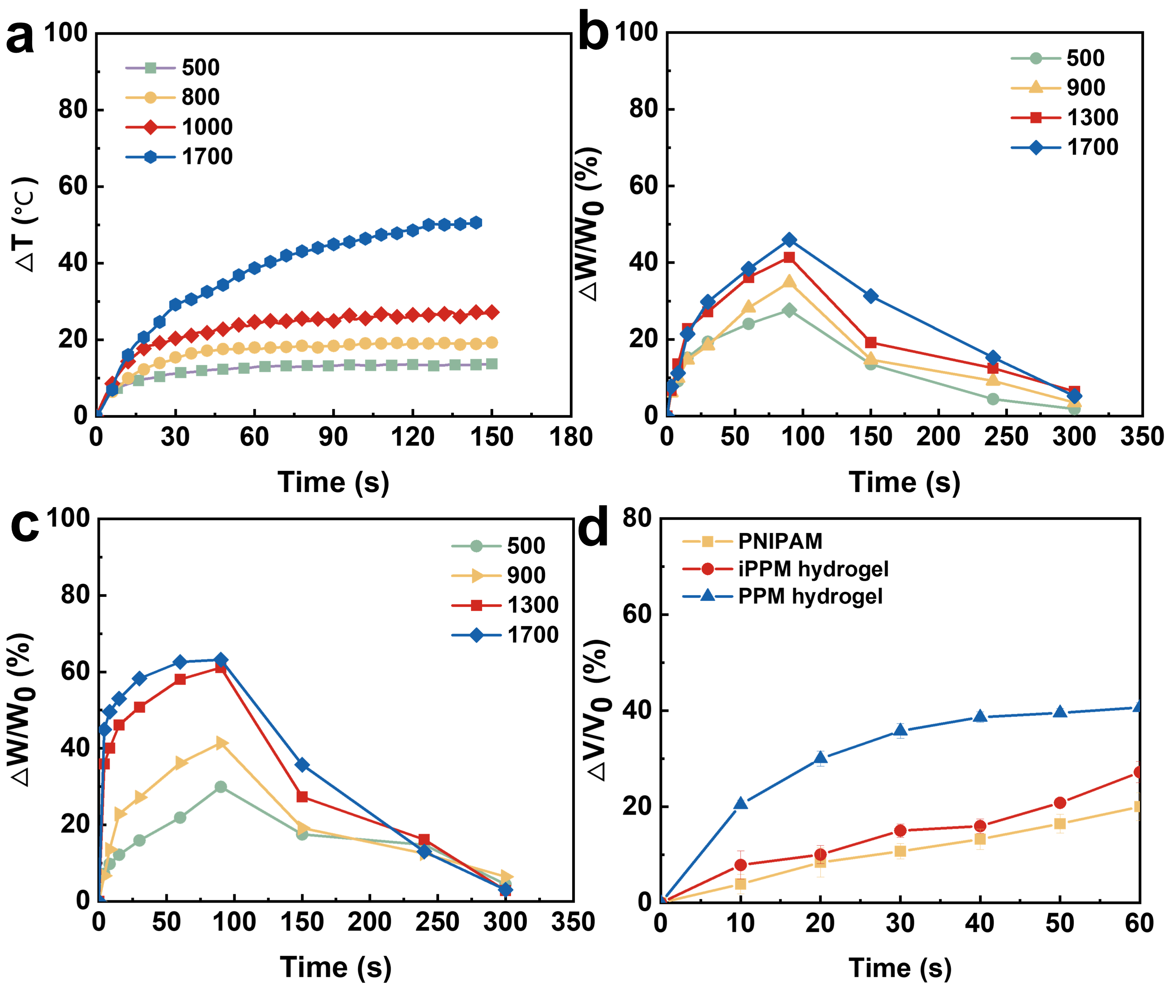


**Figure S8** a) Temperature curves of PPM hydrogel after exposure to various intensities of light; Thermal-stimulated volume shrinkage and recovery irradiated by different power densities light of b) the iPPM hydrogel and c) the PPM hydrogel; d) the deswelling rates (the temperature of the water is 40 °C) of the pure PNIPAM, iPPM hydrogel, and PPM hydrogel.

**
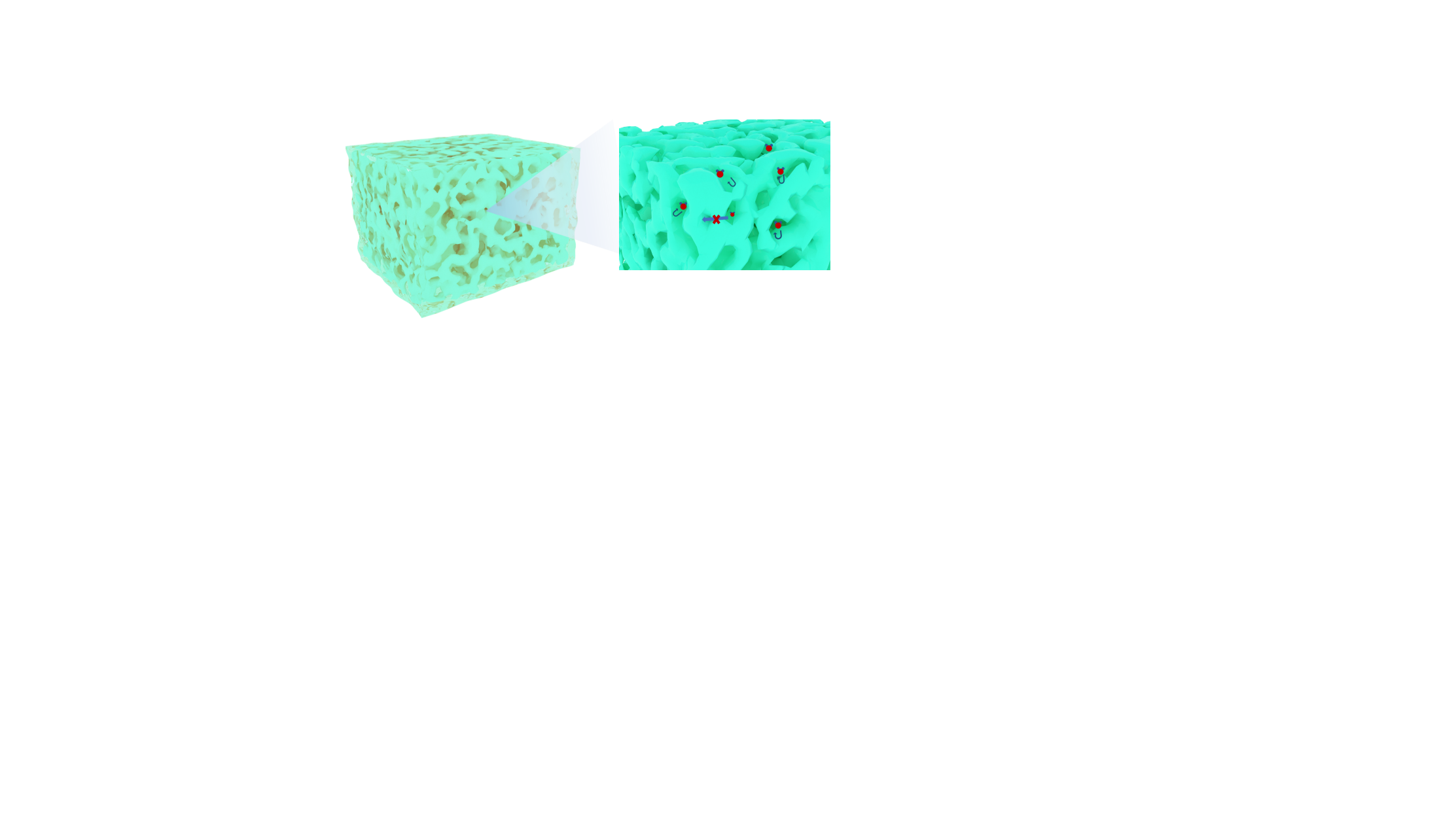
**

**Figure S9** The structural model of the isotropic hydrogel mechanism of iPPM hydrogel.


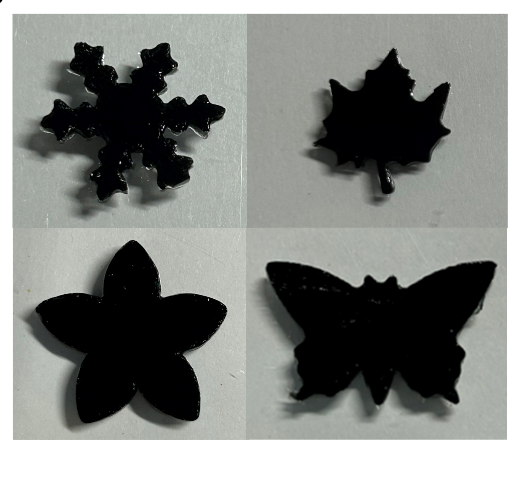


**Figure S10** The photographs of the ultra-flexible PPM hydrogel with shape adaptability.


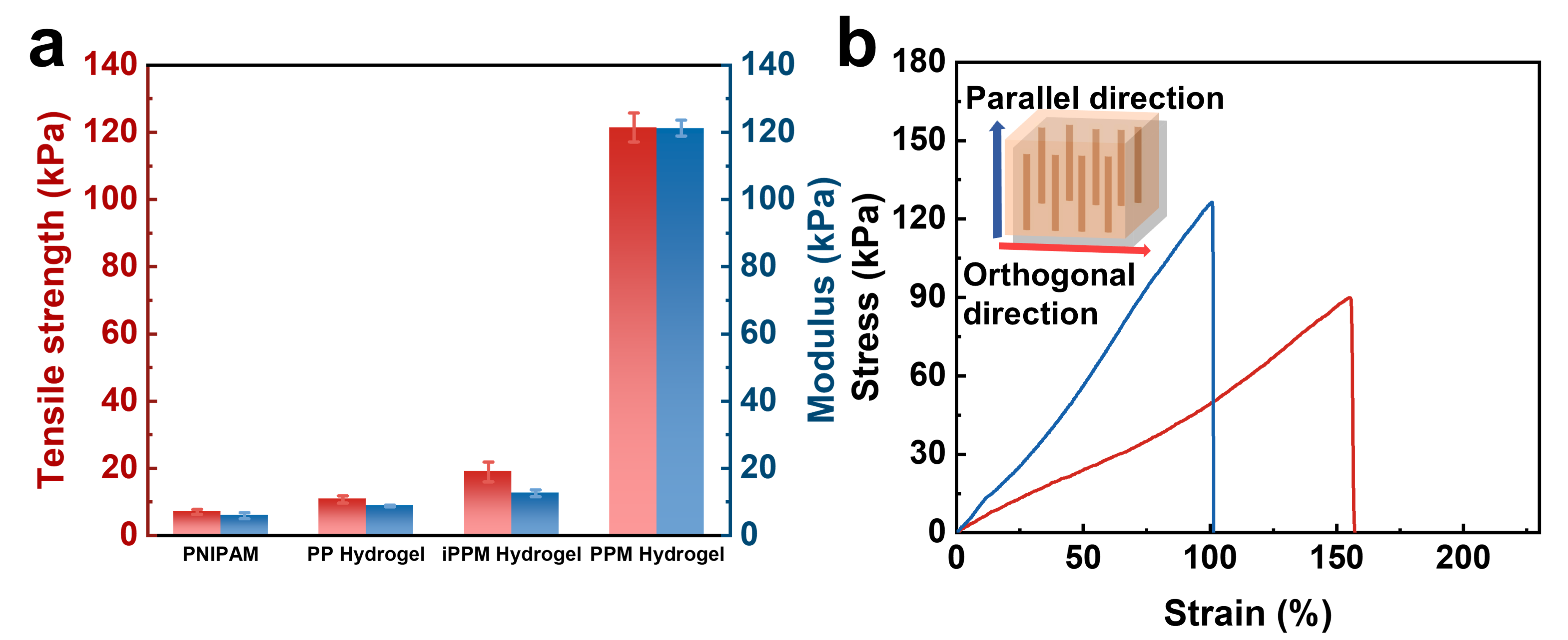


**Figure S11** a) Comparison of modulus and tensile strength of the pure PNIPAM, PP hydrogel, iPPM hydrogel and PPM hydrogel; b) The tress–strain curves of PPM hydrogel in orthogonal and parallel directions;


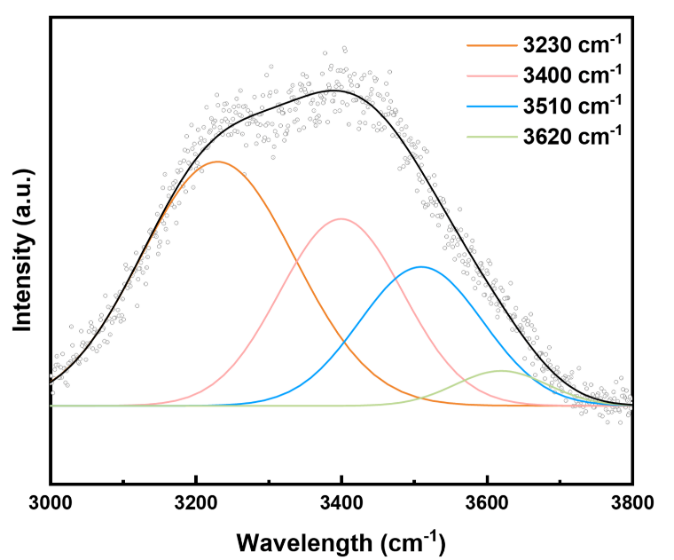


**Figure S12** Raman spectrum with fitting curves of DI water.


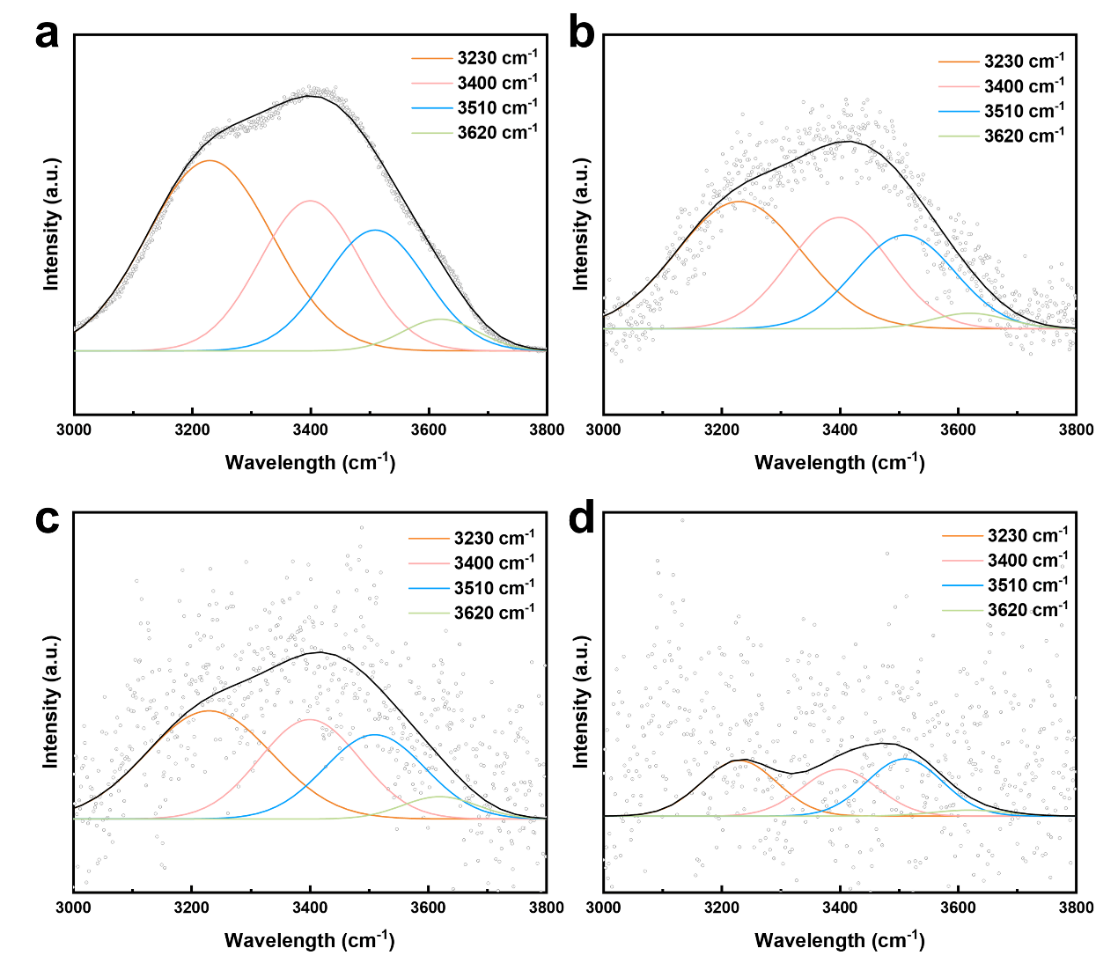


**Figure S13** Raman spectrum with fitting curves of PPM hydrogel a) at 25℃, b)45℃, c) 60℃ and d) PPM aerogel.


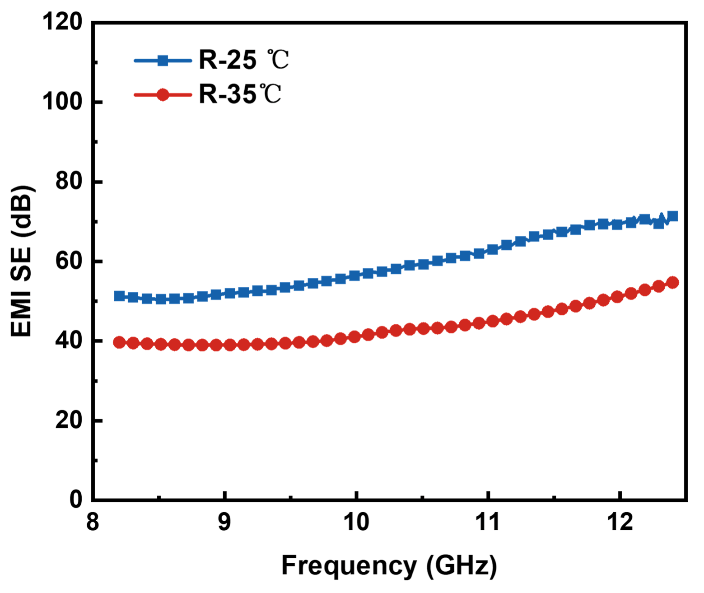


**Figure S14** The corresponding X band EMI SE curve of PPM hydrogel (with 5 wt % MXene) at different temperatures during cooling.


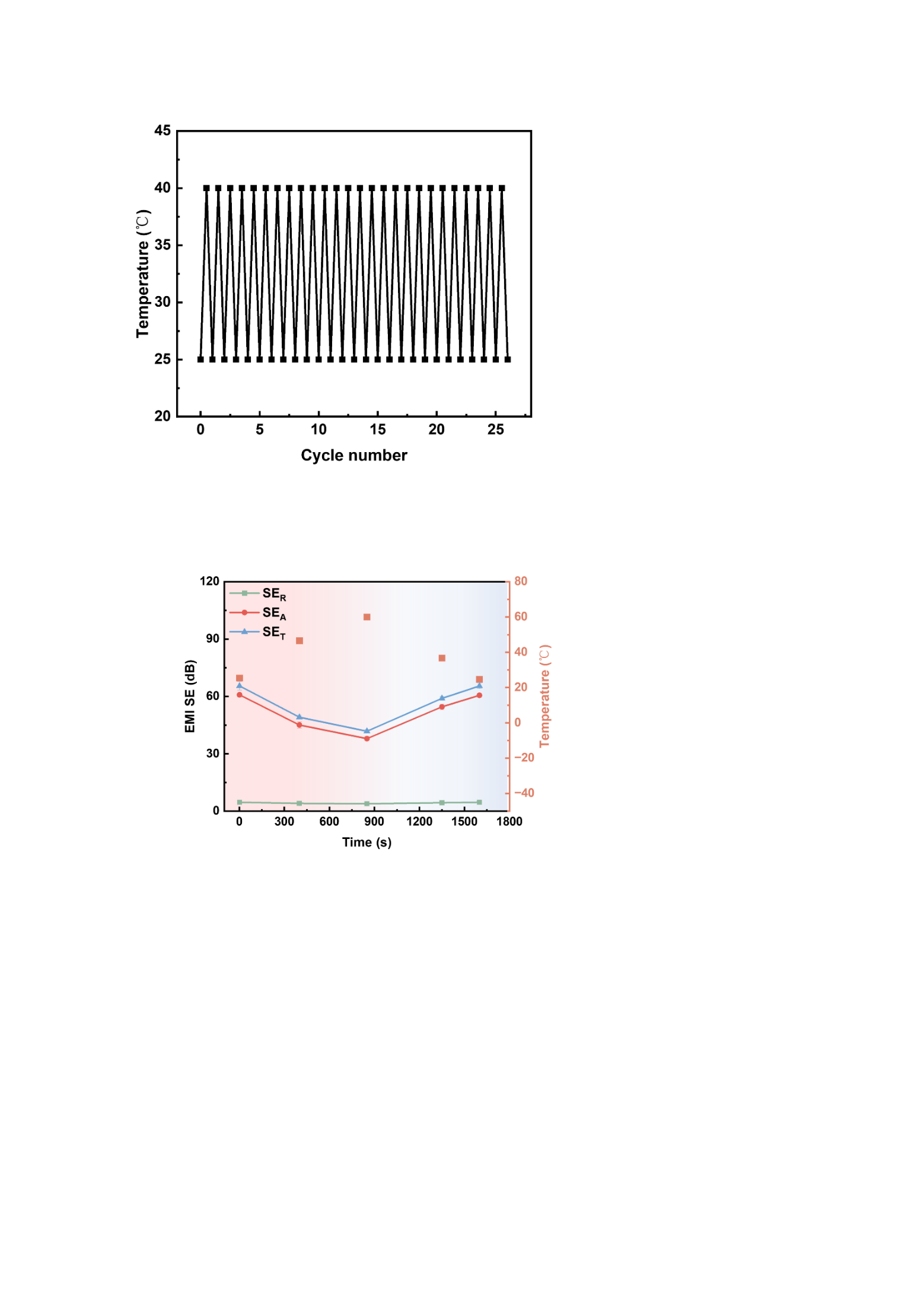


**Figure S15** Photothermal stability of PPM hydrogel.


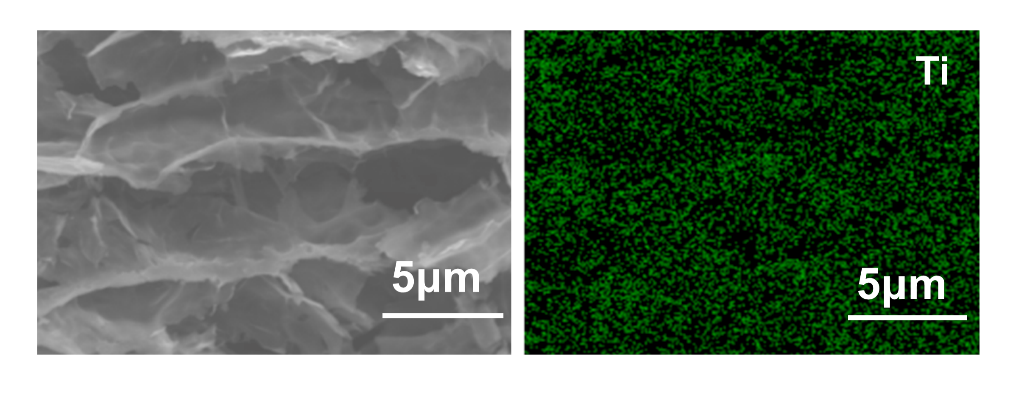


**Figure S16** SEM image of PPM hydrogel, accompanied by its corresponding EDS elemental Ti.


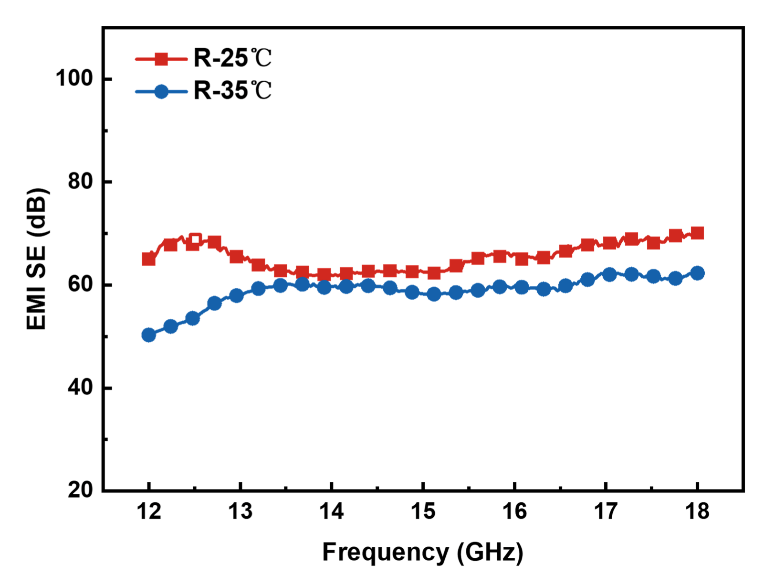


**Figure S17** The corresponding Ku-band EMI SE curve of PPM hydrogel (with 5 wt% MXene) at different temperatures during cooling.


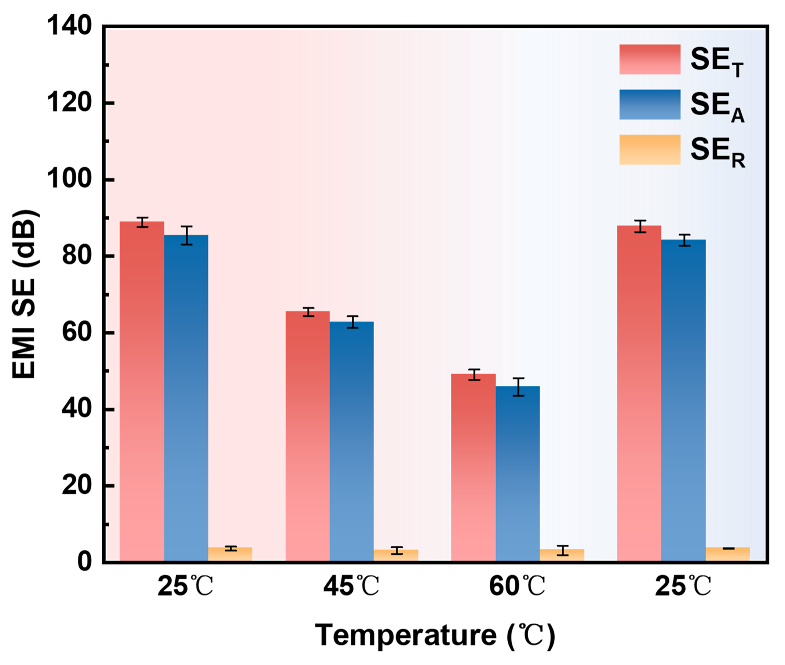


**Figure S18** The SEA, SER, and SET values in the K-band of PPM hydrogels with various temperatures.


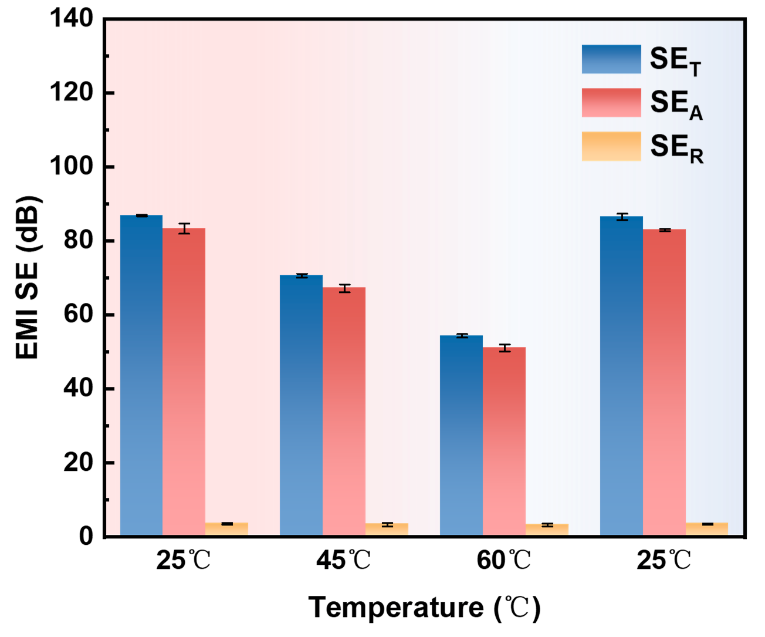


**Figure S19** The SEA, SER, and SET values in the Ka-band of PPM hydrogels with various temperatures.


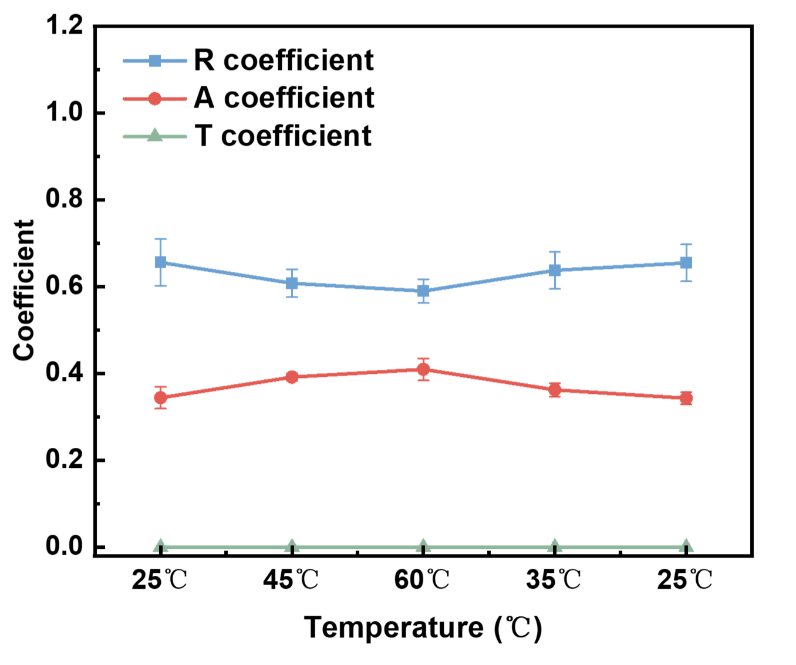


**Figure S20** The R, A, and T values of hydrogels with different temperatures in the Ku Band.


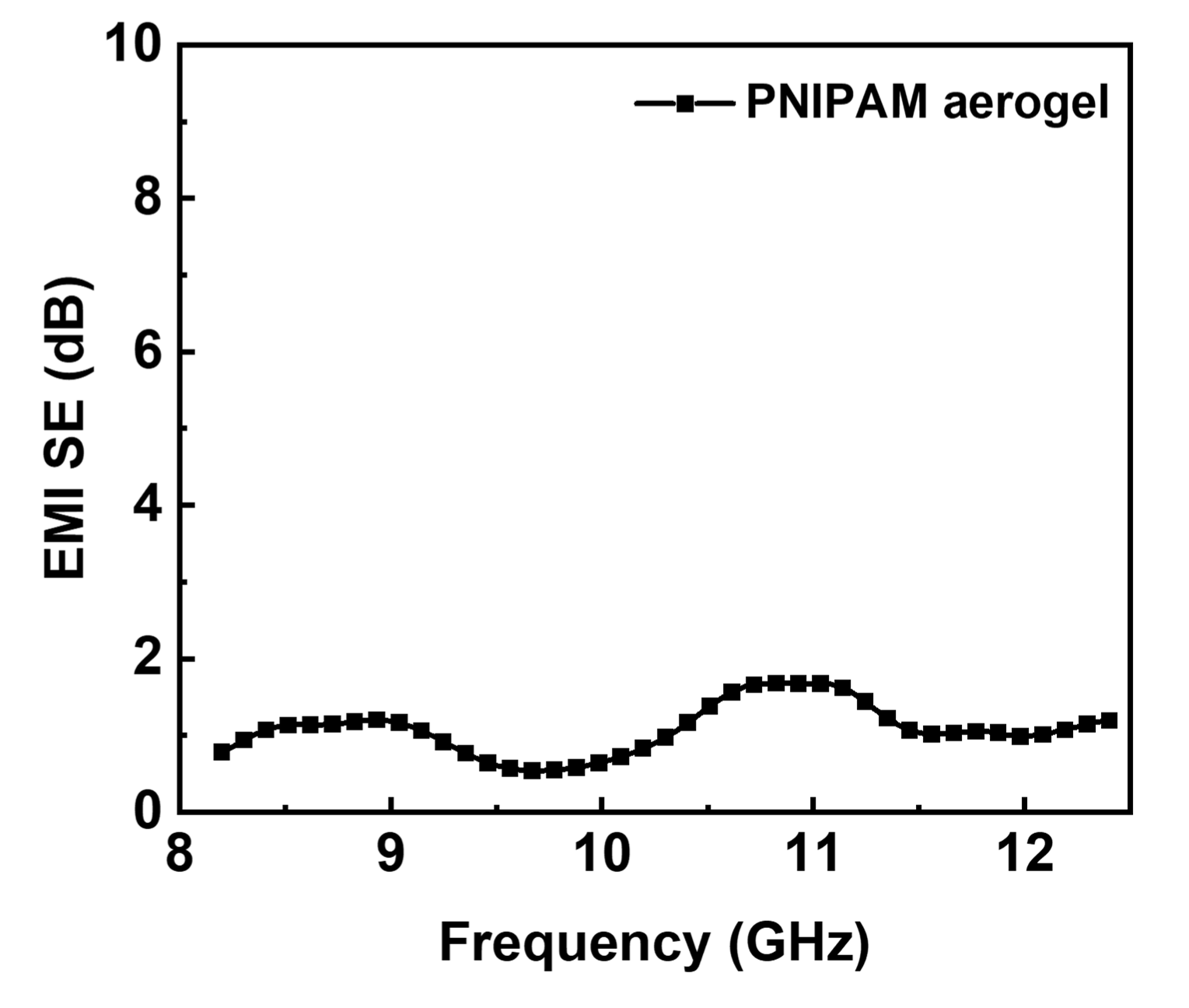


**Figure S21** EMI SE values of PNIPAM aerogels.


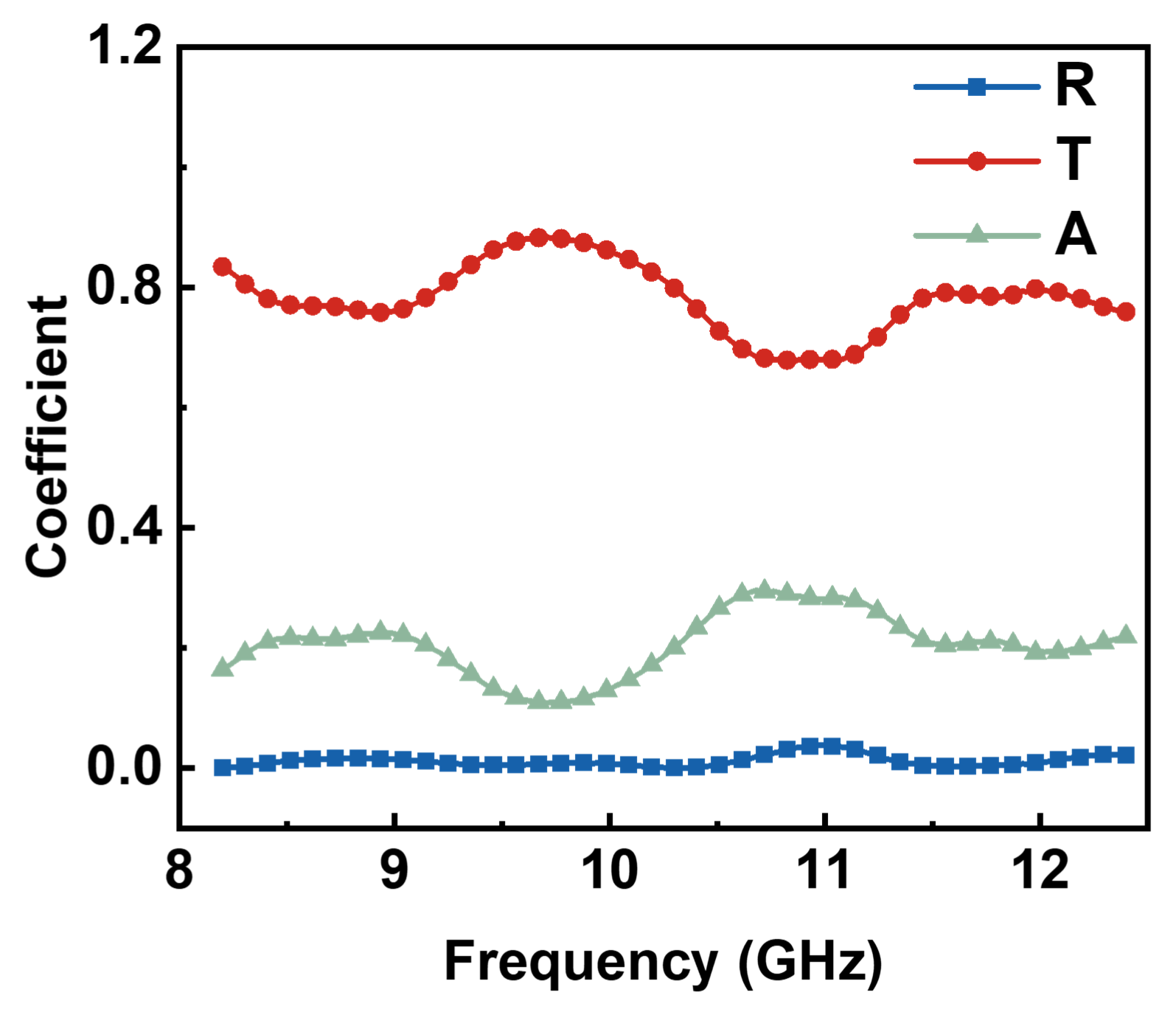


**Figure S22** The coefficient of PNIPAM aerogels.


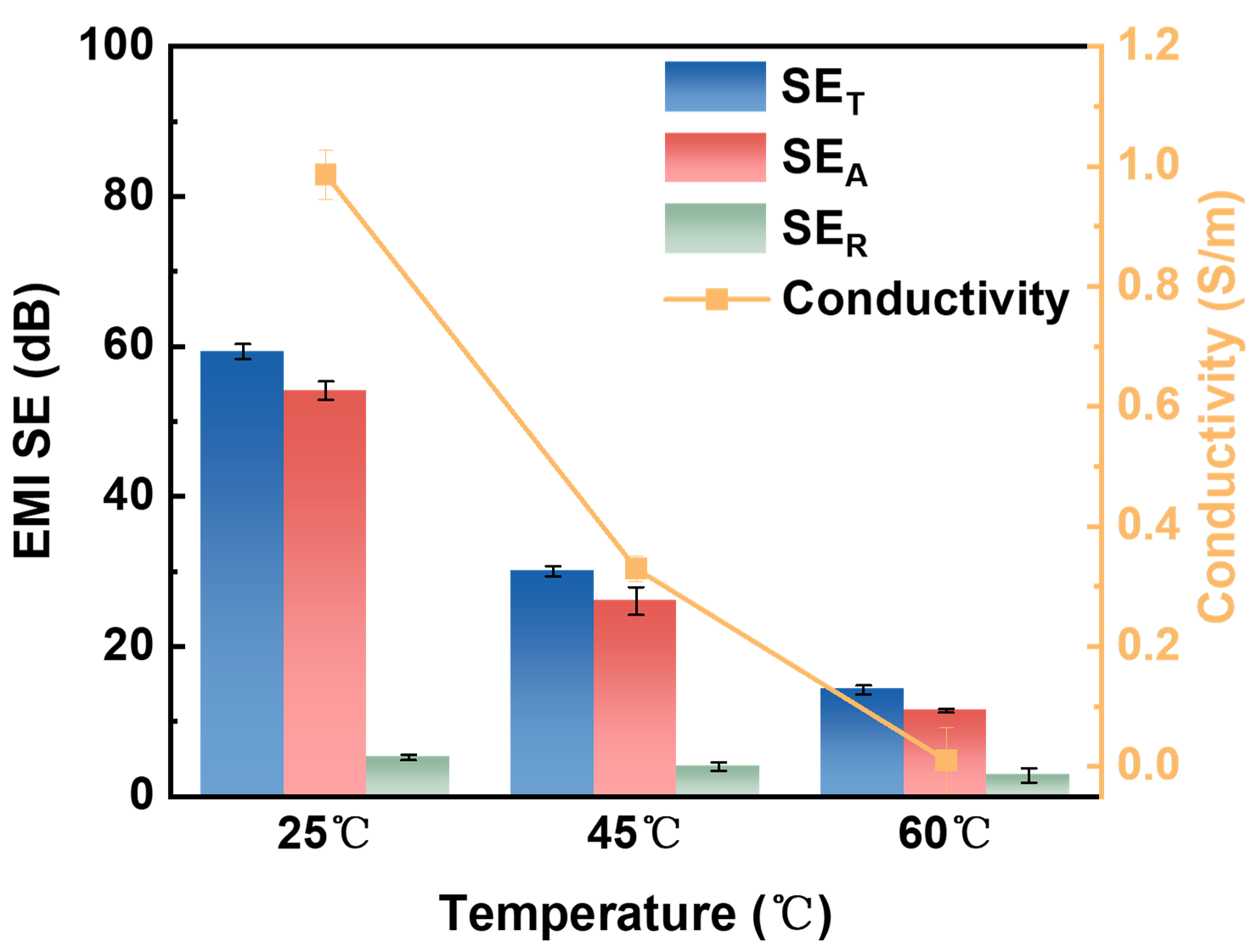


**Figure S23** The SE_A_, SE_R_, and SE_T_ values and conductivity in the X-band of PPM hydrogels with various temperatures.


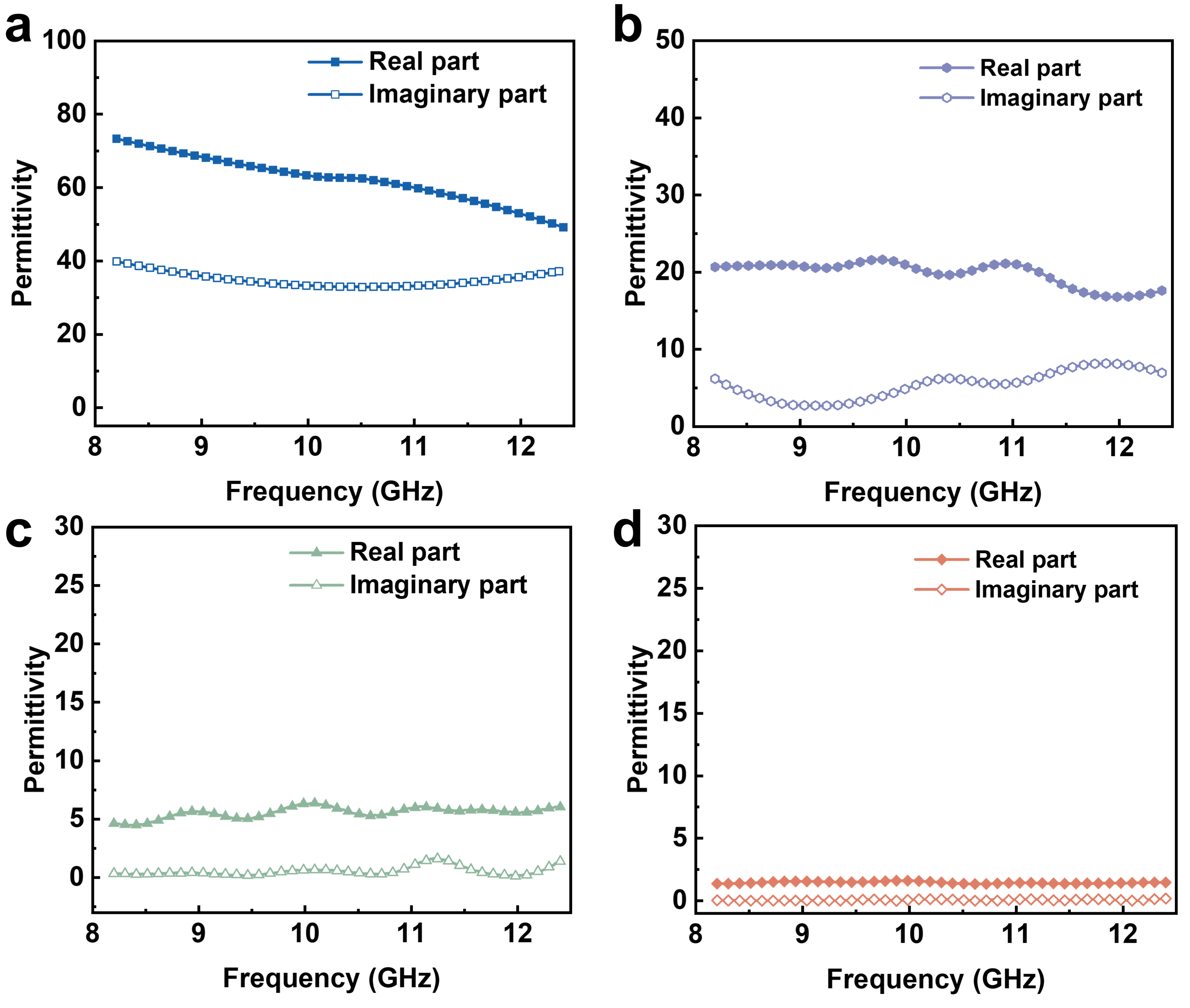


**Figure S24** Complex permittivity of hydrogels at a) 25℃, b) 45℃, c)60℃ and d) PNIPAM aerogel


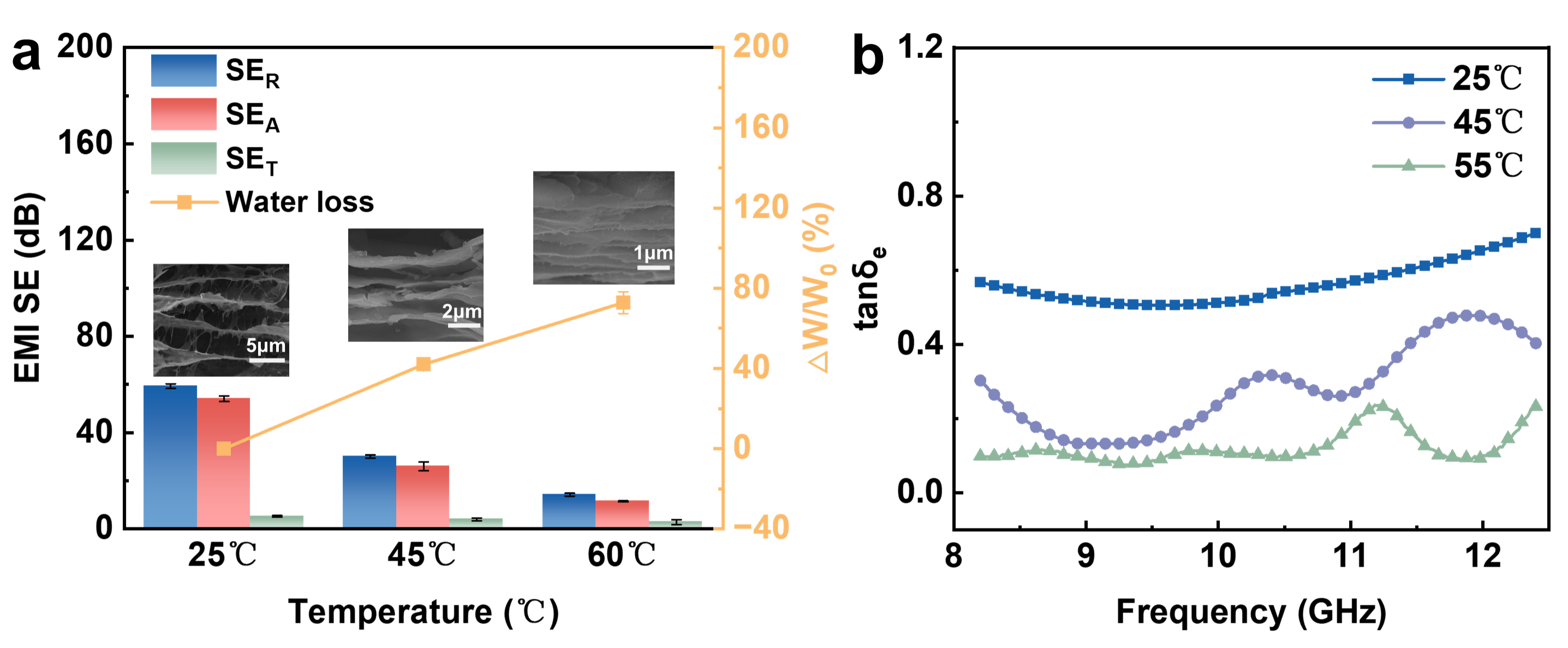


**Figure S25** a) The SE_A_, SE_R_, and SE_T_ values of PPM hydrogels in X-band and the water content and microscopic morphology with various temperatures; b) The tangent loss values of hydrogels with various temperatures.


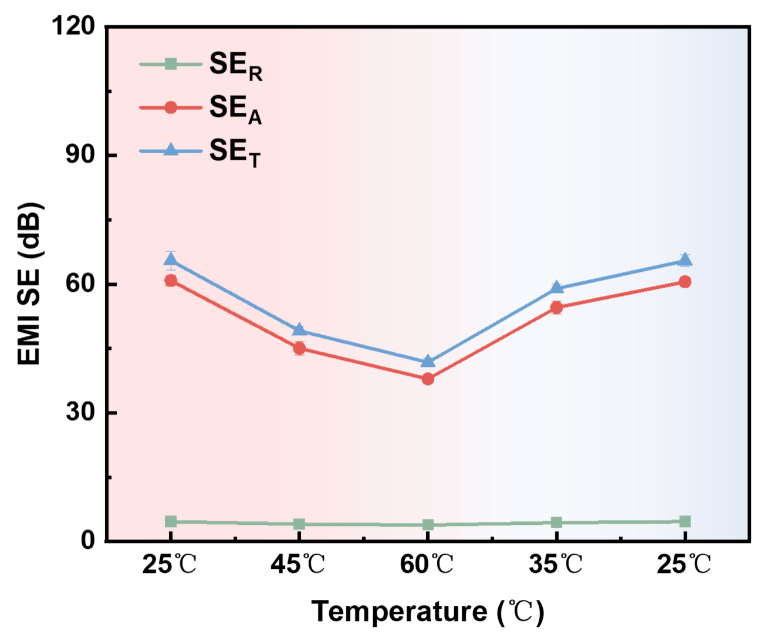


**Figure S26** SE_A_, SE_R_, and SE_T_ values in Ku-band of PPM hydrogels (with 5 wt % MXene) with various temperatures.


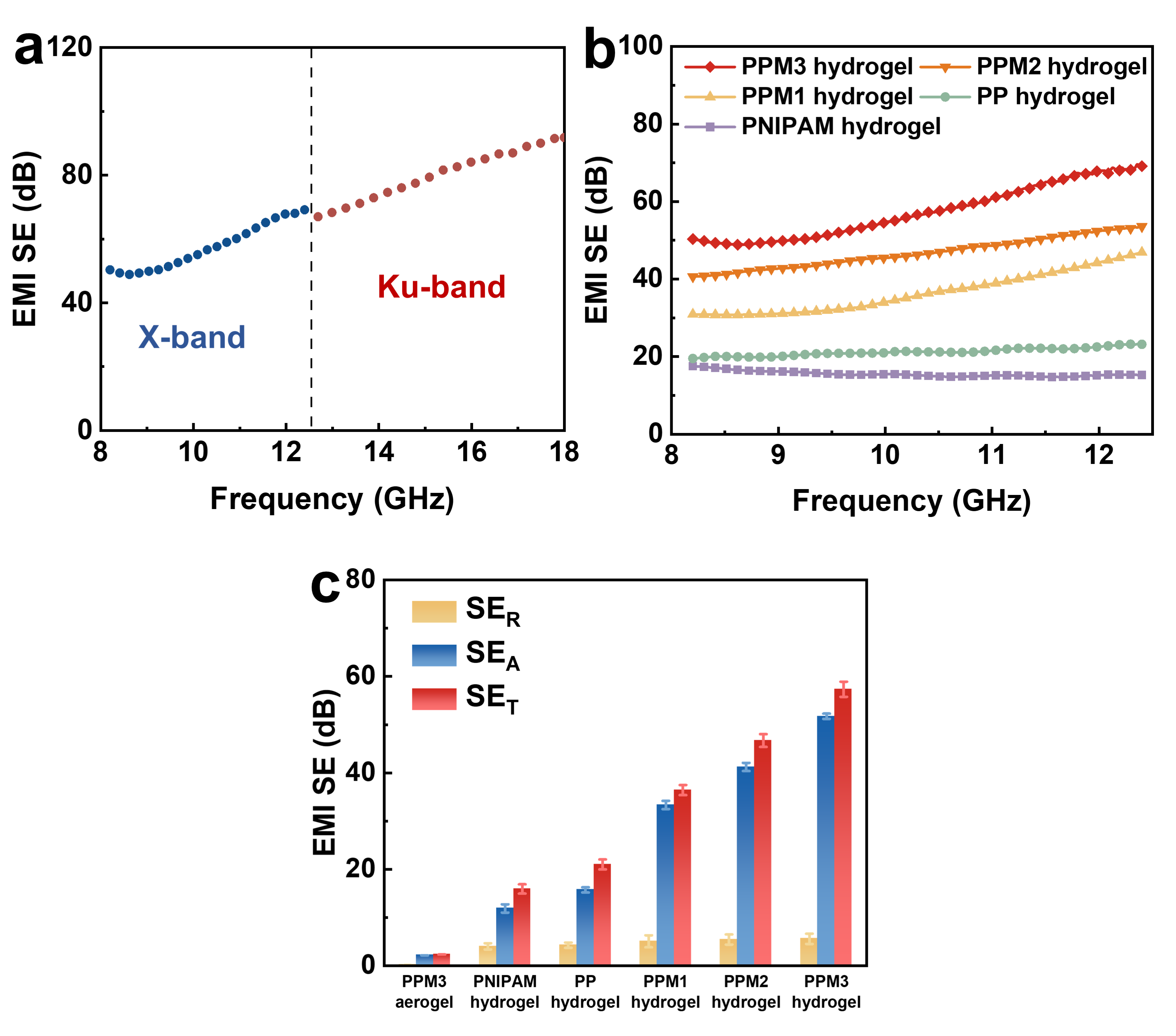


**Figure S27** a) EMI SE in the typical GHz frequency ranges including X and Ku bands. b) X-band EMI SE and c) SE_R_, SE_A_, and SE_T_ of the hydrogels with various MXene contents.


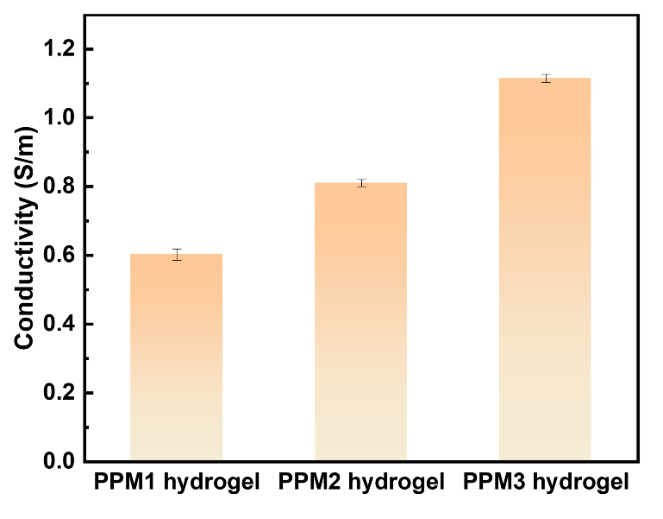


**Figure S28** The conductivity of PPM1, PPM2 and PPM3 hydrogels.


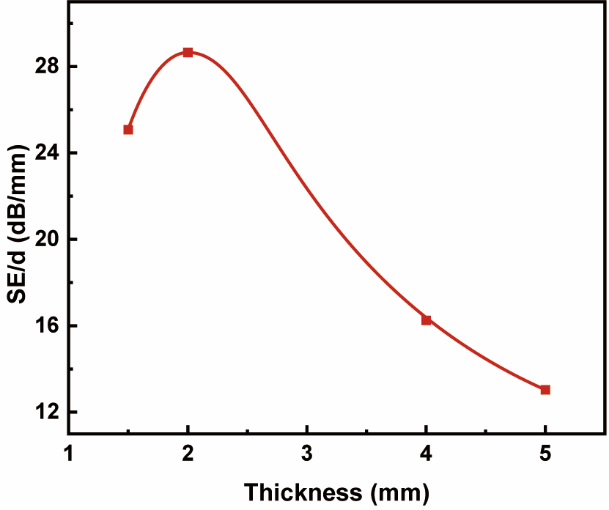


**Figure S29** Fitting curve of SE/d with PPM hydrogel thickness.


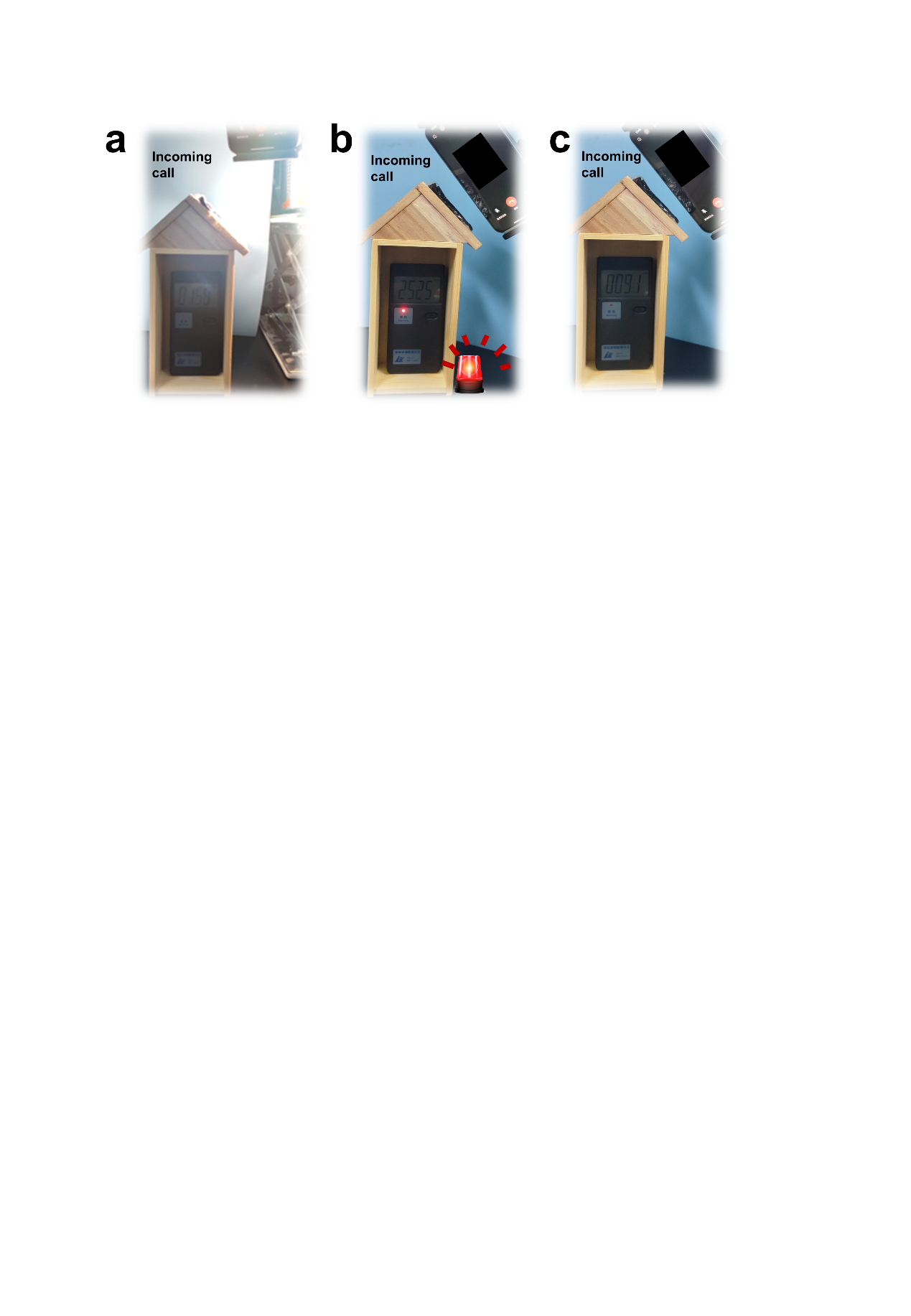


**Figure S30** Reversible electromagnetic shielding effect adjustment of PPM hydrogels on the safe house under light irradiation.


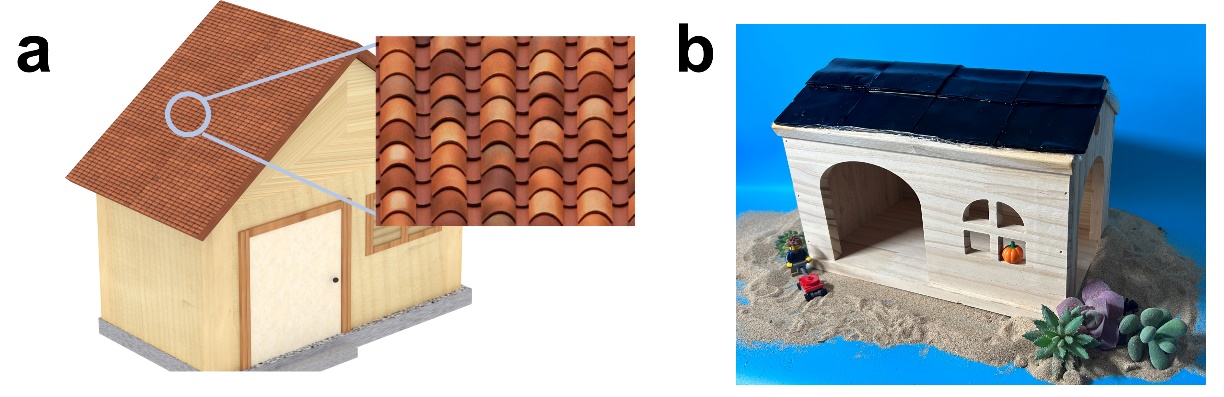


**Figure S****31** a) The schematic diagram and b) digital photo of the application of PPM hydrogel in the form of tiles in a house model.


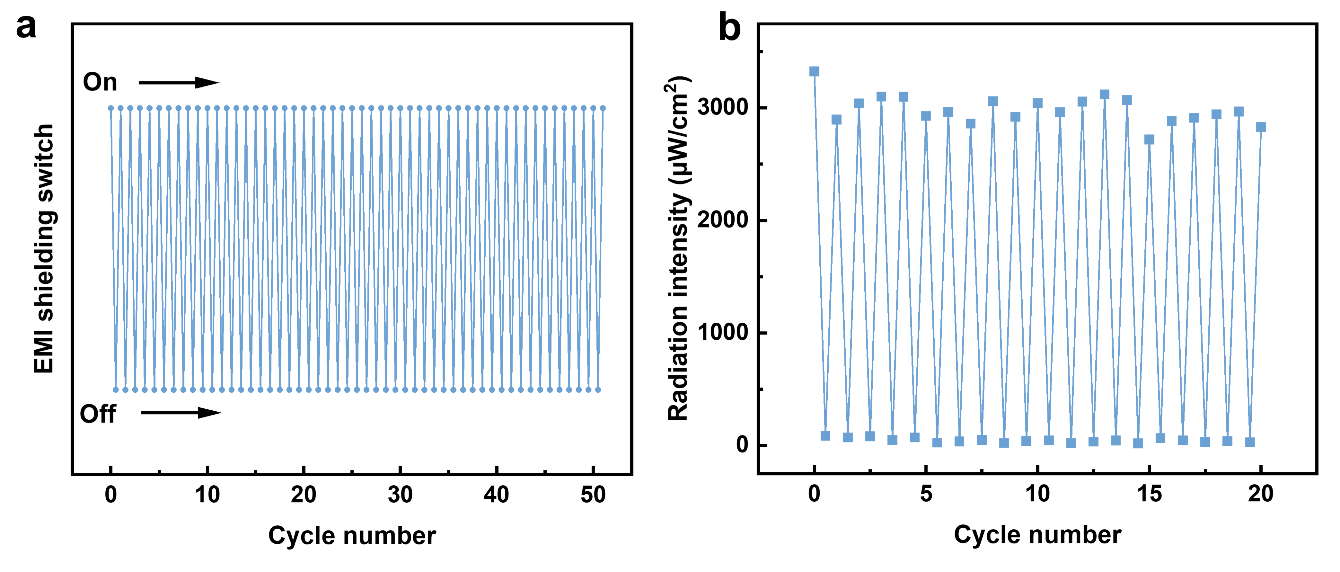


**Figure S32** The cyclic testing of the hydrogel as an intelligent shielding material switch in a) wireless charging and b) radiation power testing.

**Table S1** Comparison of the EMI shielding performance between this work and the previously reported hydrogel-based X-band shielding materials., in terms of EMI SE, response conditions, SE/d and tunable range of electromagnetic shielding parameters.

| **Samples** |  |  | **Response**  **conditions** | **EMI SE (dB)** | **Thickness (mm)** | **SE/d (dB/mm)** | **Tunable range (dB)** | **Ref** |
| --- | --- | --- | --- | --- | --- | --- | --- | --- |
| PVA/EGaInSn–8Ni hydrogel |  |  | / | 65.8 | 3.0 | 21.93 | / | ^[5]^ |
| PAM/PVA/GO/LiCl hydrogel |  |  | / | 37.2 | 3.0 | 12.40 | / | ^[6]^ |
| MXene/Ni NWs/Ni NPs/PVA-PAA hydrogel |  |  | / | 26.3 | 1.0 | 26.30 | / | ^[7]^ |
| MXene/PVA (10%MXene) hydrogel |  |  | / | 25.3 | 2.0 | 12.65 | / | ^[8]^ |
| MXene/PEDOT: PSS-Organohydrogel |  |  | / | 38.7 | 3.0 | 12.90 | / | ^[9]^ |
| Ca-PAA-CNF-MXene hydrogel |  |  | / | 25.0 | 1.5 | 16.67 | / | ^[10]^ |
| PAM/PPy/PDA hydrogel |  |  | / | 33.7 | 2.5 | 13.48 | / | ^[11]^ |
| MXene/PVA/PAA hydrogel |  |  | / | 33.6 | 2.0 | 16.80 | / | ^[12]^ |
| PPy/PVA/PA hydrogel |  |  | / | 35.0 | 2.0 | 17.50 | / | ^[13]^ |
| PAM/CNF/MWCNT hydrogel |  |  | / | 28.5 | 2.0 | 14.25 | / | ^[14]^ |
| Cellulose-based LM hydrogel |  |  | / | 33.1 | 3.0 | 11.05 | / | ^[15]^ |
| CNT@LM/PAAM/gelatin hydrogel |  |  | / | 75.7 | 4.0 | 18.92 | / | ^[16]^ |
| TAPU/Fe3O4@TA PANI organohydrogel |  |  | / | 26.0 | 2.0 | 13.00 | / | ^[17]^ |
| PEDOT:PSS/PVA hydogel |  |  | / | 41.0 | 4.5 | 9.11 | / | ^[18]^ |
| PVA/MWCNTs hydrogel |  |  | / | 123.6 | 5.0 | 24.72 | / | ^[19]^ |
| PVA/CS/MXene/Fe3O4 hydrogel |  |  | / | 49.3 | 3.0 | 16.43 | / | ^[20]^ |
| P(AM-co-AA) hydrogel |  |  | Strain (90%) | 37.0 | 2.5 | 14.80 | 37-28 | ^[21]^ |
| PAM/PNIPAM/PDMAEMA/Cu hydrogel |  |  | pH | 27.6 | 2.0 | 13.82 | 20.8-27.64 | ^[22]^ |
| MXene sediment-based hydrogel |  |  | Water | 44.7 | 2.0 | 22.35 | 44-14 | ^[4b]^ |
| PMAm/graphene hydrogel |  |  | Solvent | 32.0 | 3.0 | 10.67 | 32-21 | ^[23]^ |
| PPM hydrogel |  |  | Light & Temperature | 59.3 | 2.0 | 29.65 | 59.3-15.5 | This work |

**Reference**

[1] J. Xiong, X. Zhao, Z. Liu, H. Chen, Q. Yan, H. Lian, Y. Chen, Q. Peng, X. He, *Nanomicro Lett* **2024**, 17, 53.

[2] A. Iqbal, F. Shahzad, K. Hantanasirisakul, M.-K. Kim, J. Kwon, J. Hong, H. Kim, D. Kim, Y. Gogotsi, C. M. Koo, *Science* **2020**, 369, 446.

[3] E. Mikinka, T. Whittaker, P. Synaszko, W. Whittow, G. Zhou, K. Dragan, *Compos. Part A Appl. Sci. Manuf.* **2024**, 187.

[4] a) Y.-Q. Wang, M. Cao, B.-W. Liu, F.-R. Zeng, Q. Fu, H.-B. Zhao, Y.-Z. Wang, *Mater. Horiz.* **2024**, 11, 978; b) Y. Yang, N. Wu, B. Li, W. Liu, F. Pan, Z. Zeng, J. Liu, *ACS Nano* **2022**, 16, 15042.

[5] B. Zhao, Z. Bai, H. Lv, Z. Yan, Y. Du, X. Guo, J. Zhang, L. Wu, J. Deng, D. W. Zhang, R. Che, *Nano-Micro Lett.* **2023**, 15.

[6] C. Zhou, S. Yuan, T. Dai, S. Zhou, H. Zou, P. Liu, *Polymer* **2022**, 253.

[7] Y. Yuan, Q. You, S. Qiu, Z. Wang, Y. Chen, C. Wang, L. Zhou, H. Liu, *ACS Appl. Polym. Mater.* **2024**, 6, 11406.

[8] J. Wei, Y. Yang, F. Pan, K. Yang, Y. Wang, Z. Zeng, Q. Wang, Z. Fu, *Compos. Part A Appl. Sci. Manuf.* **2023**, 172.

[9] T.-y. Zhu, W.-j. Jiang, S. Wu, Z.-j. Huang, Y.-l. Liu, X.-d. Qi, Y. Wang, *ACS Appl. Mater. Interfaces* **2024**, 16, 15372.

[10] J. Wei, C. Zhu, Z. Zeng, F. Pan, F. Wan, L. Lei, G. Nyström, Z. Fu, *Interdisciplinary Materials* **2022**, 1, 495.

[11] A. De, A. Mondal, A. Das, P. Maity, B. B. Khatua, *J. Mater. Chem. C* **2024**, 12, 6267.

[12] Y. Xu, M. Pei, X. Zhan, J. Du, D. Zhang, *Progress in Organic Coatings* **2024**, 194.

[13] Y. Wei, T. Wu, M. Cui, Z. Gao, Z. Zhao, Y. Xue, Y. Zhang, K. Tao, J. Zhang, H. Wu, *Cell Reports Physical Science* **2024**, 5.

[14] W. Yang, B. Shao, T. Liu, Y. Zhang, R. Huang, F. Chen, Q. Fu, *ACS Appl. Mater. Interfaces* **2018**, 10, 8245.

[15] X. Feng, C. Wang, S. Shang, H. Liu, X. Huang, J. Jiang, Z. Song, H. Zhang, *Carbohydrate Polymers* **2023**, 311.

[16] H. Guo, Y. Shi, F. Pan, S. Zheng, X. Chai, Y. Yang, H. Jiang, X. Wang, L. Li, Z. Xiu, J. Wang, W. Lu, *Nano Energy* **2023**, 114, 108678.

[17] Y. Liu, Z. Zhang, X. Yang, F. Li, Z. Liang, Y. Yong, S. Dai, Z. Li, *J. Mater. Chem. A* **2023**, 11, 6603.

[18] P. Li, H. Wang, Z. Ju, Z. Jin, J. Ma, L. Yang, X. Zhao, H. Xu, Y. Liu, *ACS Nano* **2024**, 18, 2906.

[19] Y. Xu, Y. Tan, Y. Xue, M. Pei, D. Zhang, S. Liu, S. Qin, *Polymer Engineering & Science* **2023**, 63, 3555.

[20] C. Wang, L. Xu, J. Zheng, Z. Zhu, Z. Huang, C. Hu, B. Liu, *International Journal of Biological Macromolecules* **2024**, 278.

[21] Y. Lian, H. Wang, L. Liu, L. Guan, M. Lin, Q. Li, X. Guo, Z. Liu, P. Chen, B. Zhao, R. Zhang, *Small Struct.* **2024**, DOI: 10.1002/sstr.2024004862400486.

[22] X. Hu, Y. Cheng, Z. Wei, Y. Zhan, R. Zhang, H. Xia, X. Jiang, *Chem. Eng. J.* **2024**, 493.

[23] X. Xie, M. Lu, G. Li, G. Gao, J. Kang, Q. Zhang, X. Liu, *Chem. Eng. J.* **2025**, 505.
